# The reciprocal relationship between non-alcoholic fatty liver disease and hypothyroidism: A systematic review and meta-analysis of about 39 million individuals

**DOI:** 10.1371/journal.pone.0338413

**Published:** 2025-12-18

**Authors:** Ariyan Ayati Firoozabadi, Iman Elahi Vahed, Pouyan Lotfi, Adel Geshani, Ali Keshavarzian, Maryam Moftakhar, Mahkameh Razaghi, Zahra Rasouli, Mahtab Montazeri, Mohammad Ali Mansournia, Massoud Vosough, Mohammad Rahmanian

**Affiliations:** 1 School of Medicine, Shahid Beheshti University of Medical Sciences, Tehran, Iran; 2 Student Research Committee, Hamadan University of Medical Sciences, Hamedan, Iran; 3 Student Research Committee, Shiraz University of Medical Sciences, Shiraz, Iran; 4 School of Medicine, Golestan University of Medical Sciences, Gorgan, Iran; 5 School of Medicine, Ahvaz Jundishapur University of Medical Sciences, Ahvaz, Iran; 6 School of Pharmacy, Shahid Beheshti University of Medical Sciences, Tehran, Iran; 7 Student Research Committee, Kashan University of Medical Sciences, Kashan, Iran; 8 Department of Epidemiology and Biostatistics, School of Public Health, Tehran University of Medical Sciences, Tehran, Iran; 9 Department of Regenerative Medicine, Cell Science Research Center, Royan Institute for Stem Cell Biology and Technology, ACECR, Tehran, Iran; 10 Gastroenterology and Liver Diseases Research Center, Research Institute for Gastroenterology and Liver Diseases, Shahid Beheshti University of Medical Sciences, Tehran, Iran; 11 Student Research Committee, School of Medical Education and Learning Technologies, Shahid Beheshti University of Medical Sciences, Tehran, Iran; Guilan University of Medical Sciences, IRAN, ISLAMIC REPUBLIC OF

## Abstract

**Background:**

Non-alcoholic fatty liver disease (NAFLD) and hypothyroidism are both prevalent conditions with potential reciprocal influences. This study evaluates the link between NAFLD and hypothyroidism.

**Methods:**

A systematic search was performed using Scopus, PubMed, Web of Science, and Google Scholar. A random-effects meta-analysis assessed the bidirectional connection between hypothyroidism and NAFLD. Subgroup, meta-regression, and sensitivity analyses explored variability. R software (version 4.4.1) was employed for all analyses.

**Results:**

Data from 38,877,762 people were assessed, with 99% being female, limiting generalizability to male populations. Among the study population, females were significantly more numerous than males. Hypothyroidism increased the presence of NAFLD and nonalcoholic steatohepatitis (NASH) (OR = 1.96, 95% CI = 1.34–2.87; I2 = 89%). In subgroup analysis, the incidence of NAFLD/NASH was increased in both subclinical and unspecified hypothyroidism patients (OR = 1.59, 95% CI = 1.08–2.35; I2 = 91%, and OR = 1.96, 95% CI = 1.09–3.54; I2 = 79%, respectively). Conversely, overt hypothyroidism did not significantly increase the risk of NAFLD/NASH (OR = 4.27, 95% CI = 0.90–20.19; I2 = 89%). Additionally, NAFLD patients were more likely to develop hypothyroidism (OR = 1.85, 95% CI = 1.35–2.53; I2 = 100%). According to the subgroup analysis, NAFLD patients were more prone to develop subclinical hypothyroidism (OR = 1.83, 95% CI = 1.11–3.03; I2 = 87%). The increased presence of overt hypothyroidism wasn’t found to be significant in NAFLD patients (OR = 1.94, 95% CI = 0.73–5.18).

**Conclusion:**

This study suggests a reciprocal connection between hypothyroidism and NAFLD, underscoring the need for integrated management strategies and additional research into the underlying mechanisms. The predominance of female participants, combined with high heterogeneity, limits the applicability of findings to broader populations.

## Introduction

Non-alcoholic fatty liver disease (NAFLD) has become a widespread chronic liver condition, with a global prevalence of 32% and 40% among adults and males, respectively [1]. In recent years, alternative nomenclature and diagnostic frameworks have been proposed to better reflect the metabolic underpinnings of the condition. Metabolic associated fatty liver disease (MAFLD) is defined as the presence of hepatic steatosis in conjunction with at least one of the following conditions: type 2 diabetes mellitus, obesity, or metabolic dysregulation. This definition shifts the focus from excluding alcohol and other causes to emphasizing metabolic dysfunction as a central feature. In June 2023, a consensus statement from multiple medical societies was released. This statement proposed a new name for fatty liver disease, officially replacing the old term NAFLD with the new one: metabolic dysfunction-associated steatotic liver disease (MASLD). To be diagnosed with this condition, a person must have hepatic steatosis (fatty liver) confirmed by imaging or biopsy, added to at least one of the following criteria. First, the presence of obesity, defined as a body mass index (BMI) of 25 kg/m² or above (or 23 kg/m² for individuals of Asian descent) or a large waist circumference exceeding 94 cm for men or 80 cm for women, with adjustments based on ethnicity. Second, high blood sugar, defined as a fasting glucose level of 100 mg/dL (5.6 mmol/L) or more, a two-hour post-load glucose level of 140 mg/dL (7.8 mmol/L) or more, a glycated hemoglobin (HbA1c) of 5.7% or more, or current use of medication for high blood sugar. Third, increased blood pressure, defined as a blood pressure reading of 130/85 mmHg or higher, or the use of antihypertensive medication. Fourth, high triglycerides, defined as a plasma triglyceride level of 150 mg/dL (1.70 mmol/L) or more, or the use of lipid-lowering medication. Finally, low high-density lipoprotein (HDL) cholesterol, defined as a plasma HDL level below 40 mg/dL (1.0 mmol/L) for men or below 50 mg/dL (1.3 mmol/L) for women, or the use of medication intended to raise HDL cholesterol levels. This spectrum of liver disorders includes non-alcoholic steatohepatitis (NASH), cirrhosis, simple hepatic steatosis, severe fibrosis, and hepatocellular carcinoma [2]. NAFLD’s growing prevalence has been linked to the rise in metabolic syndrome, type 2 diabetes mellitus, and obesity, making it a significant public health concern [3]. Furthermore, NAFLD has a significant clinical and financial burden, as NAFLD-related liver failure is the primary cause of liver transplants in many nations [4]. Beyond its metabolic implications, NAFLD shares intricate associations with various systemic disorders [5]. Cardiovascular diseases are particularly significant, as patients with NAFLD exhibit increased risks for atherosclerosis and coronary artery disease [6,7]. Additionally, NAFLD is associated with chronic kidney disease and polycystic ovarian syndrome (PCOS), highlighting its role as a multisystemic condition [8,9]. Among these disorders, hypothyroidism stands out due to its direct impact on liver function. By disrupting metabolic homeostasis and lipid clearance, hypothyroidism contributes significantly to the progression of NAFLD [10].

As one of the most prevalent endocrine conditions in the world, hypothyroidism, with a prevalence ranging from 4% to 20%, depends on factors such as diagnostic criteria, age, and iodine intake [11,12]. This condition is not only linked to metabolic and cardiovascular complications but also associated with other systemic diseases, including depression, anemia, dyslipidemia, and infertility [13,14]. The societal burden of hypothyroidism is substantial, contributing to reduced quality of life, increased healthcare costs, and a loss of productivity [15]. Thyroxine (T4) and triiodothyronine (T3) both play essential roles in lipid metabolism regulation, insulin sensitivity, and energy production [16,17]. Evidence suggests that hypothyroidism may be linked with the initiation and exacerbation of NAFLD through mechanisms such as disrupted lipid metabolism and increased insulin resistance; however, additional research is necessary to confirm the extent of this correlation [18]. Among the factors influencing this association, body mass index (BMI) and age are particularly notable. Older age has been correlated with both an increased prevalence of hypothyroidism and greater severity of NAFLD, as metabolic regulation tends to decline with age [19,20]. Similarly, elevated BMI not only predisposes individuals to hypothyroidism through increased adiposity-related inflammation but also exacerbates hepatic steatosis and insulin resistance, thereby strengthening the connection between the two pathologic conditions [21].

By combining data from diverse studies, this meta-analysis aims to clarify the interactions between hypothyroidism and NAFLD, providing insights into associated risk factors.

## Materials and methods

This study followed the standard methodology outlined in the Cochrane Handbook [22], and the Preferred Reporting Items for Systematic Reviews and Meta-Analyses (PRISMA) 2020 guidelines were followed [23]. The study protocol has been registered in the PROSPERO database (registration ID: CRD42024604429).

### Search strategy

For this study, we thoroughly searched the following databases: PubMed, Scopus, Web of Science, and Google Scholar up to January 2025. An updated search was also performed in January 2025. A systematic approach was used, combining keywords and Medical Subject Headings (MeSH) terms to identify relevant studies. The keywords are listed as follows:

(“Hypothyroidism” OR “Hypothyroidisms” OR “Thyroid stimulating hormone deficienc*” OR “TSH deficienc*”) AND (“non-alcoholic fatty liver disease” OR “nonalcoholic fatty liver*” OR “NAFLD” OR “nonalcoholic steatohepatiti*” OR “NASH” OR “fatty liver” OR “Metabolic dysfunction–associated steatotic liver disease” OR “Metabolic dysfunction-associated fatty liver disease” OR “MASLD” OR “MAFLD”).

Additionally, forward and backward citation searching was conducted. Studies were included if they examined the connection between NAFLD and hypothyroidism, specifically investigating the impact of NAFLD on hypothyroidism, the impact of hypothyroidism on NAFLD, or a reciprocal interaction between the two conditions.

### Study selection

Two independent reviewers (A.A.F. and A.G.) assessed the eligibility of each study against predefined criteria, then both reviewed the titles, abstracts, and full texts of the studies independently, considering inclusion/exclusion criteria. The inter-rater agreement between A.A.F. and A.G. was 96%. In cases where the reviewers disagreed on whether to include or exclude a study, a third reviewer (M.R.) was consulted to make a final decision. Studies were screened based on their applicability to the study issue, focusing on the proportion of NAFLD and hypothyroidism. Age and sample size were not used as exclusion criteria. Furthermore, research was needed to define hypothyroidism and its subtypes precisely (e.g., overt and subclinical hypothyroidism), along with reliable laboratory-based diagnostic criteria. Subclinical hypothyroidism is delineated by augmented thyroid-stimulating hormone (TSH) levels, whereas thyroid hormones remain within the normal range. Overt hypothyroidism is typified by either increased serum TSH and diminished serum free thyroxine (fT4) or an exceedingly elevated TSH level (for example, 10 mU/L) [24]. All included studies had obtained ethical approval from the respective institutional review board or adhered to the Declaration of Helsinki.

Our exclusion criteria encompassed unpublished reports, letters to the editor, animal studies, conference abstracts, cellular and molecular studies, hypotheses, in vitro studies, reviews, case reports, and meta-analyses. Studies were excluded if they involved populations with pre-existing chronic liver disease (e.g., hemochromatosis, viral hepatitis, cirrhosis, Wilson’s disease, hepatocellular carcinoma) or if they included participants who did not meet the criteria for NAFLD [25]. Consequently, studies in accordance with the aforementioned inclusion criteria were those involving human populations and were observational studies.

### Data extraction

A.G. and A.A.F. developed a standard data extraction form collaboratively. Following a consensus-building process to address discrepancies, they collected and analyzed data from the studies matching the inclusion criteria. The extracted information encompassed the publication’s year, the first author’s name, the study design, the study duration, the percentage of female participants, the sample size, the country or countries of origin, the age range, and the mean age of the participants, the BMI of participants (if available), the number of cases and controls (if applicable), the criteria for case and control groups (if applicable), the incidence of NAFLD among hypothyroid patients, the incidence of hypothyroidism among NAFLD individuals, and the reciprocal relationship between NAFLD and hypothyroidism.

### Risk of bias assessment

To assess the included studies’ methodological quality and to reduce the risk of bias (RoB), a well-established tool, the Joanna Briggs Institute (JBI) [26], was employed by A.G. and A.A.F. Any discrepancy between A.A.F. and A.G. about the assessment of RoB was mediated by discussion with M.R.

### Statistical analysis

A random-effects meta-analysis was performed to estimate the odds ratio (OR) and corresponding confidence intervals (95% CI) for any potential association between hypothyroidism and NAFLD in both directions. We also estimated the pooled mean differences for TSH, free T4 (fT4), and free T3 (fT3) in the NAFLD and control groups. The restricted maximum likelihood (REML) model was applied to the analysis. Heterogeneity and inconsistency were assessed using Cochran’s Q statistics and I^2^ tests. Moreover, Egger’s regression test was conducted to evaluate possible publication bias. A *p*-value that is less than 0.05 is considered to possess statistical significance. We also performed meta-regressions and subgroup analyses for specific characteristics of the included papers to investigate potential sources of heterogeneity. To explore sources of heterogeneity between studies, meta-regression analyses were carried out using mixed-effects models with REML to estimate between-study variance (τ²). Moderator variables included the diagnostic method used for hypothyroidism or NAFLD/NASH, with ultrasonography serving as the baseline category in all models. The extent of heterogeneity explained by moderators was quantified with R². The significance of moderators was tested via the Q-test for moderators (QM). Separate meta-regression models examined five outcomes: [1] incidence of NAFLD/NASH in hypothyroid patients, [2] incidence of hypothyroidism in NAFLD/NASH patients, and [3–5] variations in serum levels of TSH, free T4 (fT4), and free T3 (fT3) among patients with NAFLD/NASH. Furthermore, sensitivity analyses were also performed to investigate the magnitude of change in the overall effect size and heterogeneity. All analyses were performed using the R software [version 4.4.1 (2024-06-14)], using the “meta” and “metafor” packages.

## Results

### Study selection

**Fig 1:**
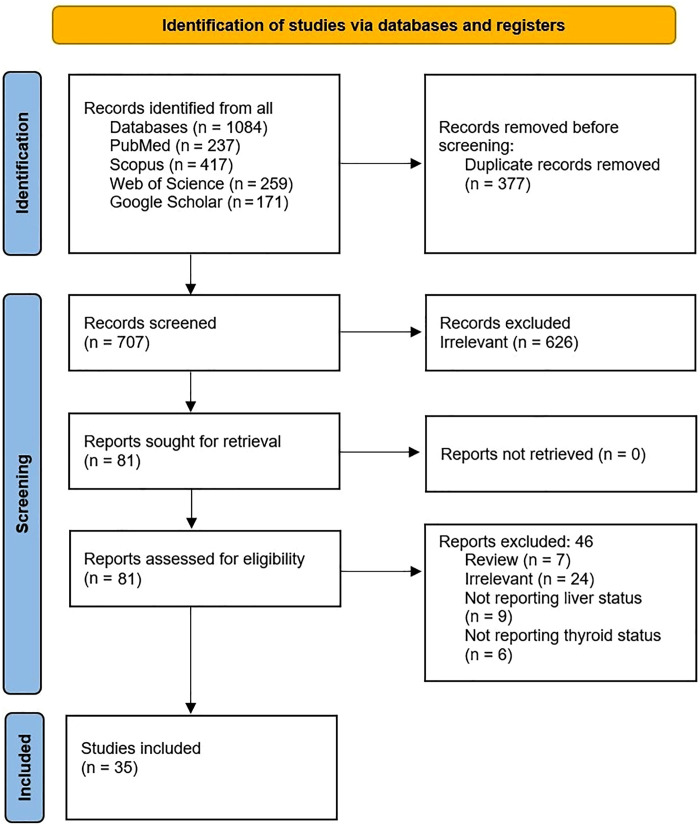
The PRISMA flowchart for inclusion of the studies.

A total of 1084 studies were identified through an advanced search using the keywords mentioned earlier in the Methods section. After duplicate removal, 707 articles entered the title and abstract screening phase, leaving 81 studies. A total of 46 studies were excluded for different reasons. For example, 24 studies were deemed irrelevant, and seven were classified as review articles. This process resulted in the inclusion of 35 articles in current study. Study selection is summarized in Fig 1.

### Study characteristics

The current study encompassed 35 observational studies. These studies were performed across 13 countries: 6 in the USA [27–32], 4 in Germany [33–36], 5 in China [37–41], 3 in Egypt [42–44], 4 in India [45–48], 2 in Iran [49,50], 3 in South Korea [51–53], 2 in Turkey [54,55], 1 in Japan [56], 1 in Mexico [57], 1 in Italy [58], 1 in the Netherlands [59], and 1 in Romania [60], and 1 was international [61]. Among these studies, 17 were cross-sectional [27,33,36,37,40,42,43,45,47–51,54,56,57,61], 11 were cohort studies [28,29,32,35,38,39,41,52,53,58,59], and 7 were case-control studies [30,31,34,44–46,60]. A total of 38,877,762 participants were assessed in our study, of whom 38,737,421 were female (equal to 99% of the total population). This high proportion of female participants is due to the study by Boustany et al., in which many females were included [32]. The mean age of participants was 51.25 years old, with a minimum of 10 years in the study by Di Sessa et al. [58] and a maximum of 69 years in the study by Tahara et al. [56]. The studies by Bano, Mahsabde, Boustany, and Loosen whether did not report age or did report stratified age structure of their study populations [32,35,48,59]. Each study’s properties are demonstrated in Table 1.

**Table 1:** Summarized characteristics and brief outcomes for each included study.

First Author, Year	Country	Study design	Population	Gender (%Female)	Follow-up duration	Hypothyroidism Definition	NAFLD detection method	Adjusted Factors	Outcome(s)	Quality score
**Bayyigit et al. 2024 [54]**	Turkey	Cross-sectional	93	60.2	½ year	SCH: TSH > 4.2 fT4 < 8.9 ng/L or TSH ≥ 10 mIU/L	US	Age, Gender, Weight, BMI, SBP, Alcohol consumption, Smoking, HGB, WBC, FBS, HDL, LDL, TG, Cr, Bilirubin	- NASH ↑ in the SCH group - In the NASH group: Age, WC, hip circumference, weight, BMI, SBP, ALT, TG, insulin, glucose, HGB, Cr, and ALP ↑ and HDL ↓	6/8
**Bi 2024 [40]**	China	Cross-sectional	284	42.6	1 year	TSH range: 0.34 to 5.60 μU/L fT4 range: 0.58 to 1.64 ng/dL	US – Liver Biopsy(Not Specified)	BMI, TG, GPT, GOT, HDL, LDL, and γ-GT	- FT3/FT4 as well as FT3 ↓ with the risk of NAFLD	7/8
**Kim et al. 2024 [53]**	South Korea	Cohort	1534	34.9	5 years	NR	US	Age, Gender, BMI, AST, ALT, TG, HDL, and LDL	- TT3 ↓ in NAFLD patients	9/11
**Kouvari et al. 2024 [61]**	Multinational	Cross-sectional	677	45.2	2 years	SCH: TSH > 4 mIU/L Normal fT3, fT4	Liver Biopsy	Age, Gender, WC, BMI, HDL, and LDL	- TSH levels ↑ in NAFLD progression	8/8
**Lu et al. 2024 [41]**	China	Cohort	432	42.8	2 years	NR	US – FLI > 60	Age, BMI, Gender, HbA1c, Smoking, Alcohol consumption, diabetes duration, diabetes medications, TG, and HDL	- Thyroid hormones and TSH were not associated with the NAFLD risk within T2DM patients.	7/11
**Mahashabde et al. 2024 [48]**	India	Cross-sectional	60	83.3	2 years	Registered	Elastography	None	- NAFLD presence ↑ in hypothyroid patients	5/8
**Wang et al. 2024 [39]**	China	Cohort	5152	54.7	1 year	SCH: TSH ≥ 4.5 mIU/L Normal fT4	US	Age, Gender, Hypertension, T2DM, TC, and TG	- In the SCH group, NAFLD ↑	9/11
**Boustany et al. 2023 [32]**	USA	Cohort	38593030	100	20 years	Registered (SNOMED-CT)	Registered (SNOMED-CT)	None	- Hypothyroidism ↑ in presence of NASH	8/11
**Disessa et al. 2023 [58]**	Italy	Cohort	2275	40.1	5 years	SCH: TSH > 4.2 mIU/L normal fT3, fT4	US – Liver Enzymes	Age, BMI, Gender	- TSH and SCH ↑ among NAFLD patients - Obesity was correlated with SCH and NAFLD	7/11
**Elshinshawy et al. 2023 [42]**	Egypt	Cross-sectional	90	87.8	1 year	SCH: TSH > 4.5 mIU/L normal fT4	Transient Elastography	Age, Gender	- In overt and SCH groups AST, ALT, GGT, insulin, HOMA-IR, TC, TG, LDL, FBS, TSH, 2HPP, and HbA1c ↑ but albumin, HDL, and fT4 ↓ - In overt participants with NASH weight, BMI, WC, insulin, HOMA-IR, and CAP measurements ↑ than without NASH. - In the SCH with NASH group, weight, BMI, WC, DBP, TSH, and HbA1c ↑ and fT4 ↓ than non-NASH.	6/8
						Overt: TSH > 4.5 mIU/L fT4 < 0.9 ng/dL				
**Fan et al. 2023 [38]**	China	Cohort	19946	47.2	6 years	SCH: TSH > 4.5 mIU/L	US	Age, Gender, BMI, Hypertension, T2DM	- In the SCH group, females, TC, HDL, LDL, TG, AST, ALP, and FBS ↑ , and ALT, GGT, and albumin ↓ compared to control. - NAFLD was not more prevalent in any group. - In the non-NAFLD group, fT4, and TSH, but not T4 ↑ compared to NAFLD group.	7/11
**Patel et al. 2023 [47]**	India	Cross-sectional	142	83.1	2 years	NR	US	Age, BMI, Hypertension, WC, HDL, TG, Alcohol consumption, Weight	- FT3, FT4, TSH, LDL, VLDL, HDL, and TC were associated with each other. - In the SCH group, NAFLD↑	6/8
**Chen et al. 2023 [27]**	USA	Cross-sectional	10445	48.4	6 years	SCH: TSH > 4.5 mIU/L	US	Age, Gender, race-ethnicity, BMI, WC, TC, HbA1c, ALT, ALP, Hypertension, DM, sedentary lifestyle, and history of cardiovascular diseases, smoking status, and alcohol consumption	- SCH was correlated with NAFLD - All-cause mortality ↑ in total and NAFLD populations - Mild SCH was linked with increased risk for cardiovascular mortality among the total and NAFLD populations - Mild SCH was associated with all-cause mortality among NAFLD patients. - Cardiovascular mortality in the total population was associated with SCH. - Severe SCH was not linked with increased mortality.	6/8
**Sheikhi et al. 2022 [50]**	Iran	Cross-sectional	926	65	2 years	SCH: TSH in range of: 4.3–10 mIU/L normal fT4 and fT3 levels	US	Age, Gender, BMI, BP, DM, lipids, smoking, and alcohol consumption	- The Mean Age, BMI, FBS, HbA1C, and DM duration of diabetic NAFLD patients ↑ compared to non-NAFLD patients. - SCH and not overt hypothyroidism prevalence ↑ in NAFLD group - the confounding effect of Age, Gender, BMI, and NAFLD risk was 2.32 times ↑ in SCH than in non-SCH patients	6/8
						Overt TSH ≥ 10 mIU/L Low fT3 and fT4				
**Labenz et al. 2021 [34]**	Germany	Case-control	114966	52.3	15 years	ICD-10 code	Not Reported	Age, Gender, Obesity, DM	- The hypothyroid group, ↑ NAFLD, and the 18–50-year-old Age group showed the greatest impact	10/10
**Loosen et al. 2021 [35]**	Germany	Cohort	81164	50	15 years	ICD-10 code	Not Reported	Age, Gender, Obesity, DM	- In the hypothyroid group NAFLD risk ↑	7/11
**Grewal et al. 2020 [45]**	India	Cross-sectional	200	81.5	1.5 year	Elevated serum TSH	US – Liver enzymes	Age, Gender	- NAFLD risk ↑ in cases with BMI < 25, hypothyroidism, ↑ cholesterol - NAFLD risk did not differ between obese patients of the two groups.	5/8
**Kim et al. 2020 [29]**	USA	Cohort	10144	47.3	23 years	SCH: TSH > 4.5 mIU/L normal FT4	US	Gender, ethnicity, BMI, WC, smoking habits, DM, Hypertension, ALT, TC, HDL, history of cardiovascular disease, economic status, and sedentary lifestyle	- The NAFLD prevalence ↑ with increasing TSH - SCH was associated with ↑ NAFLD risk. - All-cause mortality ↑ in SCH and NAFLD comorbid patients	9/11
**Popescu et al. 2020 [60]**	Romania	Case-control	248	73.4	NR	SCH: TSH > 4.5 mIU/L normal FT4	US	Age, Gender	- NAFLD and metabolic syndrome were significantly correlated with hypothyroidism and its grade. - The WC, BMI, FBS, ALT, AST, TG, and BP showed differences between the euthyroid and hypothyroid subjects. - NAFLD ↑ with metabolic syndrome. - NAFLD featured with high ALT was significantly more prevalent among hypothyroid patients - Hypothyroid patients had ↑ levels of ALT compared to euthyroid ones.	6/10
**Tahara et al. 2019 [56]**	Japan	Cross-sectional	140	48.6	6 years	SCH: TSH > 4 mIU/L normal FT4	US	BMI, TG, HDL, Hypertension, and DM	- NAFLD was significantly correlated with SCH, Age, Gender, and BMI. - TSH and not fT4 levels were independently ↑ with NAFLD - TSH rise was ↑ with the fibrosis progression.	7/8
**Assem et al. 2018 [43]**	Egypt	Cross-sectional	90	50	¼ year	NR	US	Age, Gender	- AST, ALT, TC, HOMA-IR and TG were correlated in both NAFLD and non-NAFLD. - TSH was correlated with Age in the NAFLD patients. - Significant association was observed between NAFLD and SCH.	5/8
**Kim et al. 2018 [28]**	USA	Cohort	425	47.8	4 years	SCH: TSH > 4.5 mIU/L normal FT4	Liver Biopsy	Age, Gender, BMI, smoking, Hypertension, and DM, TC, TG, HOMA-IR	- Advanced liver fibrosis and NASH ↑ in SCH patients.	7/11
**Bano et al. 2016 [59]**	Netherlands	Cohort	5200	56.5	10 years	SCH: TSH ≥ 4 mIU/L Normal fT4	US	Age, smoking, Gender, alcohol intake, TC, TG, BMI, Hypertension, follow-up time, and DM	- The risk of NAFLD and cardiovascular risk factors ↓ with fT4 level ↑ . - NAFLD presence was correlated with TSH level. - NAFLD risk was elevated in hypothyroid patients.	7/11
						Overt: TSH ≥ 4 mIU/L fT4 < 10.9 pmol/L				
**Gokmen et al. 2016 [55]**	Turkey	Case-control	115	65.2	½ year	SCH: TSH ≥ 4.1 mIU/L	US	Age, Gender	- WC, TG, BMI, SBP, and DBP levels ↑ in NAFLD patients. - ET patients with NAFLD had ↑ WC, TC, TG, HOMA-IR, and fasting insulin versus non-NAFLD	7/10
**Kaltenbach et al. 2016 [33]**	Germany	Cross-sectional	332	51.2	1 year	SCH: TSH > 4 mIU/L normal FT4	US	Age, BMI-SDS, and Stage of puberty	- In the NAFLD group, BMI, HOMA-IR, SBP, TSH, AST, ALT, and GGT were elevated. - TSH levels were correlated with hepatic steatosis	6/8
**Kassem et al. 2016 [44]**	Egypt	Case-control	120	66.7	1 year	SCH: TSH ≥ 4.2 mIU/L	US	Age, Gender	- ALT, GGT, FBS, HbA1C, TC, LDL, HDL, TG, and TSH ↑ in NAFLD patients - fT3 and fT4 ↓ within NAFLD patients - TSH was associated with ALT, GGT, HbA1C, TG, fT4, and fT3 among NAFLD cases.	6/10
**Ding et al. 2015 [37]**	China	Cross-sectional	1154	20.3	5 years	SCH: TSH > 5.30 mIU/L Overt: TSH > 5.30 mIU/L fT4 < 7.86 pmol/L	Liver Biopsy	Age, Gender	- NAFLD was not correlated with hypothyroidism. - fT3 and TSH in CHB and NAFLD patients ↑ versus non-NAFLD and CHB patients. - TSH level ↑ in hepatic steatosis - fT3 was correlated with male gender - T3 level ↑ with intensity of hepatic inflammation and ↓ with Age - Serum HBV content and liver inflammation severity were correlated with NAFLD and ↓ with NASH.	6/8
**Lee et al. 2015 [52]**	South Korea	Cohort	18544	46.7	5 years	SCH: TSH ≥ 4.2 mIU/L Normal fT4	US	Gender, Age, BMI, TG, and HDL	- Overt hypothyroidism and SCH were not correlated with NAFLD but ↑ with metabolic syndrome.	8/11
						Overt: TSH ≥ 3.4 mIU/L fT4 < 0.97 ng/dL				
**Ludwig et al. 2015 [36]**	Germany	Cross-sectional	1276	47.2	1 year	SCH: TSH ≥ 3.4 mIU/L Normal T3, T4	US	Age, BMI	- T4 ↓ was associated with NASH ↑	6/8
						Overt: TSH ≥ 3.4 mIU/L total T4 < 12.8 pmol/L				
**Parikh et al. 2015 [46]**	India	Case-control	800	64.9	2 years	Thyroid replacement therapy or former diagnosis	Liver Biopsy	Age, Gender, and BMI	- NAFLD and NASH ↑ with hypothyroidism - Age, sex, and TG levels were the same in hypothyroid and ET NAFLD cases. - AST, ALT, and BMI ↑ with hypothyroidism.	6/10
**Posadas-Romero et al. 2014 [57]**	Mexico	Cross-sectional	753	54.1	4.5 years	SCH: rised TSH normal fT4	Abdominal CT-Scan	Age, BMI, Gender, fT4, LDL, and hs-CRP	- Fatty liver was not associated with TSH quartiles. - hs-CRP and non-HDL-C were the only differences between SCH and ET fatty liver patients. - fatty liver patients demonstrated ↑ BMI, WC, TC, HDL, FBS, HOMA-IR, ALT, and AST compared to euthyroid patients. - Fatty liver in euthyroid ↑ metabolic syndrome and insulin resistance.	6/8
**Chung et al. 2012 [51]**	South Korea	Cross-sectional	4648	62.4	3 years	SCH: TSH > 4.1 mIU/L normal fT4	US	Age, Gender, BMI, WC, TG, HDL, TC, Hypertension and DM	- NAFLD and changed ALT and AST ↑ in correlation with hypothyroidism intensity, independently. - NAFLD was associated with NAFLD risk in every BMI group. - ↓ fT4 was a risk factor for NAFLD. - Dose-dependent analysis showed that NAFLD was correlated with TSH.	6/8
						Overt: fT4 < 0.7 ng/dL				
**Eshraghiyan et al. 2013 [49]**	Iran	Cross-sectional	832	61.3	1 year	SCH: TSH > 5.2 mIU/L Normal fT4	US		- NAFLD was not correlated with a change in thyroid hormones and TSH. - Hypothyroidism was correlated with NAFLD	6/8
						Overt: TSH > 5.2 mIU/L fT4 < 11.5 pmol/L				
**Padagala et al. 2011 [31]**	USA	Case-control	909	56.4	2.6 years	Levothyroxine replacement therapy + former diagnosis	Liver Biopsy	DM, Hypertension, BMI, and Hyperlipidemia	- Both NAFLD and NASH were associated with ↑ hypothyroidism risk. - mild alcohol consumption was inversely correlated with hypothyroidism. - Hypothyroidism was also correlated with ↑ risk of NAFLD and NASH. - Hypothyroidism was correlated with older age, female Gender, and ↑ BMI among NAFLD patients.	8/10
**Liangpunsakul et al. 2003 [30]**	USA	Case-control	616	59	6 years	Levothyroxine replacement therapy + former diagnosis	Liver Biopsy	Age, Gender, Race, Weight, Comorbidities (DM, Hypertension, and Hyperlipidemia)	- NASH was associated with ↑ risk of hypothyroidism. - Hypothyroidism was ↑ in NASH when compared with the liver disease controls.	7/10

**Symbols:**  ↑ : increased, ↓ : decreased; **Abbreviations:** WC: waist circumference, BMI: body mass index, (S/D) BP: (systolic/diastolic) blood pressure, HGB: Hemoglobin, WBC: white blood cell, TC: total cholesterol, HDL: High-density lipoprotein, LDL: Low-density lipoprotein, TG: serum triglycerides, Cr: creatinine, (T2) DM: (Type 2) diabetes mellitus, FBS: fasting blood glucose, ALT: Alanine transaminase, AST: Aspartate transferase ALP: Alkaline phosphatase, GGT: Gamma-glutamyltransferase HbA1C: Hemoglobin A1C, TSH: Thyroid-stimulating hormone (f/t) T4: (free/total) Thyroxine, fT3: free triiodothyronine, HOMA-IR: Homeostatic Model Assessment for Insulin Resistance, hs-CRP: high- sensitivity C-reactive protein, SCH: Subclinical hypothyroidism, ET: Euthyroid, US: Ultrasonography, SNOMED-CT: Systematized Nomenclature of Medicine Clinical Terms, FLI: Fatty Liver Index.

### Quality assessment

According to the JBI critical appraisal checklists for observational studies, 17 included cross-sectional studies (S1 Table), 11 cohort studies (S2 Table), and 7 case-control studies (S3 Table) had a mild to low risk of bias, all of which fulfilled the required score for inclusion.

### Data syntheses

#### Assessment of NAFLD/NASH in hypothyroid patients.

**Fig 2:**
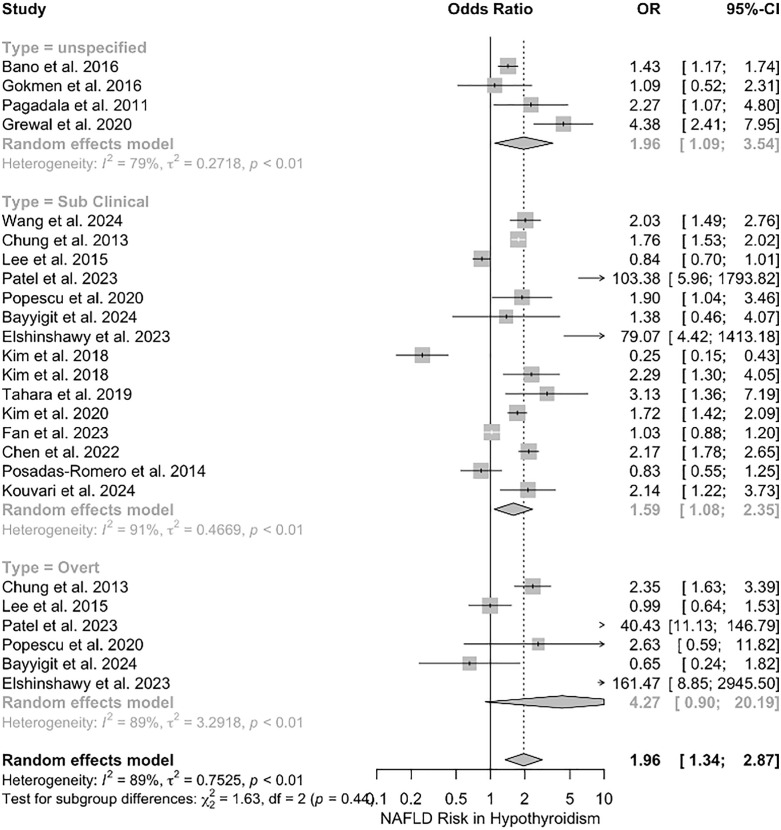
Forest plot depicting the association between hypothyroidism subtypes (subclinical hypothyroidism [SCH], overt hypothyroidism [OH], and unspecified hypothyroidism [UH]) and the odds of NAFLD or NAFLD. Odds ratios (OR) with 95% confidence intervals (CI) are presented, with heterogeneity assessed by I².

A total of 18 studies evaluated the NAFLD incidence in the presence of hypothyroidism. Hypothyroidism was significantly associated with an elevated presence of NAFLD/NASH (OR = 1.96, 95% CI = 1.34–2.87; I2 = 89%). (Fig 2). A significant publication bias and asymmetric funnel plot were observed (*p*-value < 0.0001) (S1 Fig). We also conducted a sensitivity analysis to identify potential sources of heterogeneity; however, no individual study was recognized as the source. Subgroup analysis was also performed based on the hypothyroidism subtype, NAFLD or NASH subtypes, study design, and study location to reduce heterogeneity.

Subclinical and unspecified hypothyroidism were both linked with a higher incidence of NAFLD/NASH significantly (OR = 1.59, 95% CI = 1.08–2.35; I2 = 91%, and OR = 1.96, 95% CI = 1.09–3.54; I2 = 79%, respectively). In contrast, overt hypothyroidism did not have any correlation with NAFLD/NASH incidence (OR = 4.27, 95% CI = 0.90–20.19; I2 = 89%) (Fig 2).

Hypothyroidism was also linked with significantly higher odds of both NAFLD and NASH (OR = 1.8, 95% CI = 1.16–2.79; I2 = 91% and OR = 3.16, 95% CI = 1.08–9.23; I2 = 71%) (S2 Fig). Based on the study designs, the cross-sectional (OR = 3.8, 95% CI = 1.7–8.53; I2 = 85%) and case-control (OR = 1.76, 95% CI = 1.20–2.58; I2 = 0%) studies suggested a significant rise in the presence of NAFLD/NASH, in contrast with the cohort studies (OR = 1.15, 95% CI = 0.71–1.85; I2 = 90%) (S3 Fig).

Another subgroup analysis performed was based on the study location. It showed that NAFLD is significantly more prevalent in a hypothyroid context in Romania (OR = 1.99, 95% CI = 1.14–3.47; I2 = 0%), Egypt (OR = 112.71, 95% CI = 14.57–871.99; I2 = 0%), and India (OR = 19.54, 95% CI = 2.98–128.27; I2 = 84%) but not in the USA, Turkey, China, and South Korea (S4 Fig).

The last subgroup analysis was performed based on the method used for detecting NAFLD/NASH. NAFLD was more incident among hypothyroid patients when detected with ultrasonography (US) (OR = 1.9, 95% CI = 1.36–2.66; I2 = 89%) and transient elastography (TE) (OR = 112.71, 95% CI = 14.57–871.99; I2 = 0%), in contrast to liver biopsy evaluation (OR = 1.28, 95% CI = 0.43–3.78; I2 = 93%) (S5 Fig).

#### Assessment of hypothyroidism in NAFLD/NASH patients.

A total of 15 studies evaluated the incidence of hypothyroidism in patients with a history of NAFLD/ NASH. The analysis indicated that NAFLD/NASH patients had a higher incidence of hypothyroidism (OR = 1.85, 95% CI = 1.35–2.53; I2 = 100%). We also performed a sensitivity analysis to explore potential sources of heterogeneity; however, no single study was identified as being responsible for the observed heterogeneity. In the sensitivity analysis, after leaving the study by Boustany et al. out [32], including around 38 million female participants, the association was still significant (OR = 1.58, 95% CI = 1.24–2.02). Subgroup analyses were performed based on hypothyroidism classification, study design, and study location. Publication bias and funnel plot asymmetry were also assessed via Egger’s test, which resulted in a symmetrical funnel plot (p-value = 0.0512) (S6 Fig).

**Fig 3:**
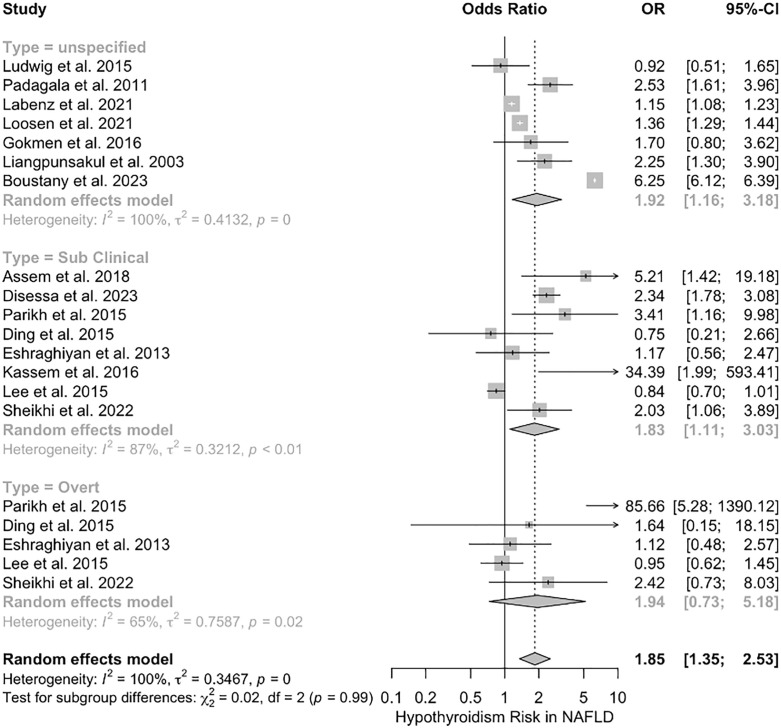
Forest plot depicting the association between NAFLD or NASH and the risk of hypothyroidism subtypes (SCH, OH, UH). OR with 95% CI are shown, with heterogeneity evaluated using I².

According to the results of the subgroup analysis, subclinical hypothyroidism was significantly higher in NAFLD/NASH patients (OR = 1.83, 95% CI = 1.11–3.03; I2 = 87%). The analysis of unspecified subgroup (studies not clarifying the type of hypothyroidism) also revealed a significant presence of hypothyroidism among NAFLD patients (OR = 1.92, 95% CI = 1.16–3.18; I2 = 100%). However, overt hypothyroidism was not significantly higher in NAFLD patients (OR = 1.94, 95% CI = 0.73–5.18; I2 = 65%) (Fig 3).

Classified based on the study design, the analysis of case-control studies showed a significant elevation of hypothyroidism detection (OR = 2.35, 95% CI = 1.41–3.90; I^2 ^= 83%), yet the increase in the analyses of cross-sectional and cohort studies was not meaningful (OR = 1.42, 95% CI = 0.98–2.05; I^2 ^= 25% and OR = 1.75, 95% CI = 0.85–3.62; I^2 ^= 100%) (S7 Fig).

Regional analysis showed that the incidence of hypothyroidism in the NAFLD context was significantly higher in Germany (OR = 1.23, 95% CI = 1.05–1.44; I^2 ^= 88%), the USA (OR = 3.43, 95% CI = 1.76–6.7; I^2 ^= 93%), Egypt (OR = 8.60, 95% CI = 1.68–44.05; I^2 ^= 82%), Iran (OR = 1.54, 95% CI = 1.04–2.30; I^2 ^= 0%), and Italy (OR = 2.34, 95% CI = 1.78–3.08; I^2 ^= 0%) as opposed to India, China, and South Korea (S8 Fig).

Based on the NAFLD/NASH detection method, hypothyroidism was more significantly incident among both US- and liver biopsy-based subgroups (OR = 1.47, 95% CI = 1.06–2.05; I2 = 82%, and OR = 2.40, 95% CI = 1.75–3.30; I^2 ^= 51%, respectively) (S9 Fig).

#### Assessment of the alterations of thyroid-related hormones in NAFLD patients.

Concentrations of three hormones were also assessed in NAFLD patients: free T3 (fT3), free T4 (fT4), and thyroid-stimulating hormone (TSH). fT3 and fT4 levels were reported in 10 studies. At first, neither fT3 nor fT4 showed a significant alteration in NAFLD patients (MD = 0.07, 95% CI = −0.09–0.23; I^2 ^= 86% and MD = −0.93, 95% CI = −2.29–0.42; I^2 ^= 96%) (S10 and S11 Figs). However, the sensitivity analysis identified the study by Kassem *et al.* (2016) as a source of influence on the heterogeneity of the overall outcomes in fT4 analysis. Excluding this study, the I2 value decreased from 97% to 47%, yet the results remained unchanged. The levels of fT4 were still insignificantly lower in NAFLD patients (MD = −0.18, 95% CI = −0.44–0.08; I^2 ^= 47%) (S12 Fig). Publication bias was not present among the included studies in these two contexts. Therefore, their funnel plots were symmetrical (the *p*-values for fT3 and fT4 were 0.0829 and 0.4930, respectively) (S10 and S11 Figs). Among subgroup analysis outcomes (based on NAFLD detection method), no subgroup showed alteration in either fT3 or fT4 levels (S13 and S14 Figs).

**Fig 4:**
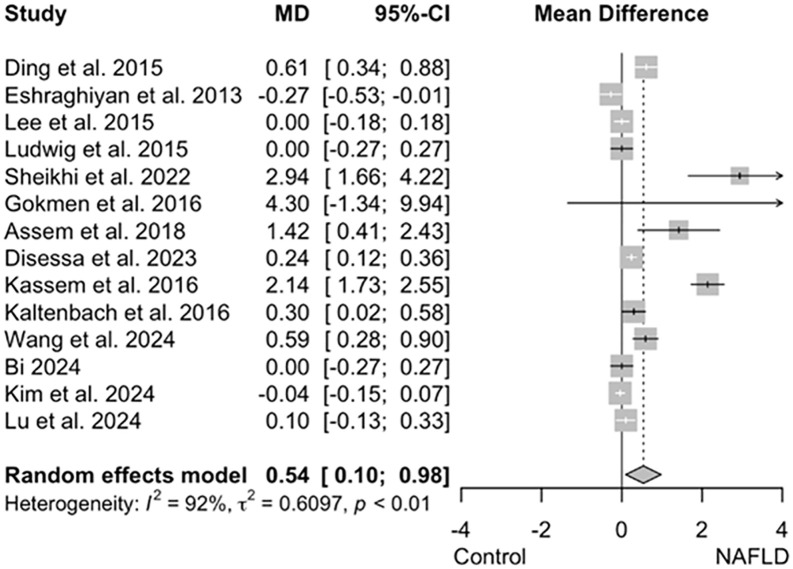
Forest plot showing TSH levels in NAFLD patients compared to controls, post-sensitivity analysis excluding the Mahashabde study. Mean difference (MD) with 95% CI is presented, with heterogeneity assessed by I².

Finally, 14 of the included studies assessed TSH levels in patients with NAFLD. Initially, our analysis revealed that TSH levels were increased insignificantly in the presence of NAFLD (MD = 2.97, 95% CI = −1.28–7.22; I^2 ^= 95%) (S15 Fig). However, after sensitivity analysis, the study by Mahashabde was removed, and subsequently the result was altered, and heterogeneity decreased by 3%; the TSH increase was proved significant in NAFLD patients (MD = 0.54, 95% CI = 0.10–0.98; I^2 ^= 92%) (Fig 4). Publication bias was proved to be significant, and the funnel plot was asymmetrical (p-value < 0.0001) (S15 Fig). Subgroup analysis outcomes based on NAFLD detection method showed that TSH levels in the US subgroup are slightly increased (MD = 0.61, 95% CI = 0.07–1.14; I^2 ^= 93%) (S16 Fig).

All sensitivity analyses data for the risk of hypothyroidism in the context of NAFLD (S4 Table), the risk of NAFLD in the context of hypothyroidism (S5 Table), the fT3 level in the context of NAFLD (S6 Table), the fT4 level in the context of NAFLD (S7 Table), and the TSH level in the context of NAFLD (S8 Table) are available within the supplementary material.

#### Meta-regression.

Meta-regression analysis was conducted to explore the potential influence of age, BMI, and gender on the five previously specified comparisons: NAFLD risk in hypothyroid patients, hypothyroidism risk in NAFLD patients, fT3, fT4, and TSH levels in NAFLD patients.

The analysis revealed significant correlations in certain factors, as detailed below. BMI was directly linked to the risk of hypothyroidism in NAFLD individuals, meaning that higher BMI levels correlated with an increased risk of developing hypothyroidism in NAFLD patients. Additionally, female gender was associated with a higher risk of a bidirectional relationship between NAFLD and hypothyroidism, as well as TSH changes in NAFLD patients. The overall results of this meta-regression analysis are shown in Table 2.

**Table 2:** P-values from meta-regression analysis examining the correlation of age, BMI, and female gender with the risk of NAFLD in hypothyroid patients, hypothyroidism in NAFLD patients, and thyroid hormone levels (fT3, fT4, TSH) in NAFLD patients.

Risk of NAFLD in Hypothyroid Patients	
**Age**	0.9407
**BMI**	0.2763
**Sex**	**<.0001**
**Risk of hypothyroidism in NAFLD patients**	
**Age**	0.5479
**BMI**	**0.0045**
**Sex**	**<.0001**
**fT3 levels in NAFLD patients**	
**Age**	0.4821
**BMI**	0.3944
**Sex**	0.0577
**fT4 levels in NAFLD patients**	
**Age**	0.6497
**BMI**	0.0684
**Sex**	0.1139
**TSH levels in NAFLD patients**	
**Age**	0.9284
**BMI**	0.6695
**Sex**	**0.0083**

#### QM meta-regression.

All five models exhibited substantial residual heterogeneity (I² = 87.8% to 98.8%), indicating the need for QM meta-regression based on the study-level diagnosis method for NAFLD. While evaluating NAFLD/NASH prevalence in hypothyroid patients, the diagnostic method was not a significant moderator (QM = 1.62, p = 0.4448; R² = 0.0%). The intercept (US) showed a significant effect (p = 0.0429), whereas liver biopsy and unspecified methods did not significantly differ from ultrasonography. When assessing the incidence of hypothyroidism among NAFLD/NASH patients, the diagnostic method significantly explained part of the heterogeneity (QM = 14.17, p = 0.0027; R² = 22.7%). The US served as the reference method, while transient elastography showed a significantly stronger association (p = 0.0007). Other methods, such as liver biopsy and CT scan, did not significantly differ from US. In TSH alterations, the diagnostic method was highly significant (QM = 97.71, p < 0.0001; R² = 98.9%). Transient elastography was repeatedly associated with a significantly higher effect size compared to US (p < 0.0001), whereas liver biopsy and unspecified methods showed no notable difference. For fT4 and fT3, none of the diagnostic methods significantly moderated the results (p > 0.5), and R² was near zero. The intercept (US) in these models was not statistically significant. The results of QM meta-regression are presented in Table 3.

**Table 3:** QM meta-regression outcomes for the association between NAFLD/NASH and hypothyroidism, including number of effect sizes (k), heterogeneity (I²), proportion of heterogeneity explained (R²), test for moderators (QM p-value), significant moderators, and ultrasonography (US) effect p-value.

Outcome	k	I² (%)	R² (%)	QM (*p*-value)	Significant moderator(s)	US effect (*p-*value)
**NAFLD/NASH in hypothyroid patients**	20	98.81	0.00	0.4448	None	0.043
**Hypothyroidism in NAFLD/NASH patients**	25	95.79	22.67	0.0027	Transient Elastography	0.0008
**TSH changes in NAFLD patients**	15	98.26	98.89	0.0001	Transient Elastography	0.026
**fT4 changes in NAFLD patients**	10	98.41	0.00	0.8320	None	0.176
**fT3 changes in NAFLD patients**	9	87.75	0.00	0.516	None	0.870

**Symbols:** k: number of effect sizes included for each analysis, I²: Observed between-study heterogeneity, R²: The proportion of between-study heterogeneity explained by the moderator, QM p-value: the result of the test for moderators indicating at least one significant moderator.

## Discussion

The current meta-analysis, encompassing 35 studies, discussed that primary hypothyroidism significantly correlates with higher NAFLD risk. Certain links between NAFLD/NASH and subclinical and unspecified hypothyroidism were identified. However, overt hypothyroidism did not significantly correlate with the NAFLD prevalence. Hypothyroidism is also correlated positively with higher incidence rates of NAFLD and NASH. The regional analysis also indicated that the occurrence of hypothyroidism in NAFLD patients was elevated in Germany, the USA, Egypt, Iran, and Italy as compared to India, China, and South Korea. Among other factors, the occurrence of hypothyroidism among NAFLD patients was positively linked to BMI, whereas age did not demonstrate any correlation. Finally, female gender was correlated with the bidirectional association between NAFLD and hypothyroidism and TSH changes in NAFLD individuals.

Liangpunsakul et al. were the first to show a meaningful prevalence of hypothyroidism in NASH individuals against a control group in 2003 [62]. Subsequently, multiple studies addressed this association; for example, Xu et al. carried out a study with 327 adult participants diagnosed with subclinical hypothyroidism and 327 controls, and their evaluation showed a strong link between subclinical hypothyroidism and a higher odds of developing NAFLD [63]. Kim et al. found that individuals with low grades of hypothyroidism displayed a dose-dependent association with NAFLD [29]. A meta-analysis by Mantovani et al. demonstrated that primary hypothyroidism was linked with an elevated prevalence of NAFLD [64]. Bano et al., in a prospective cohort involving 9,419 elderly individuals with normal thyroid function [65], discovered that any hypothyroidism was independently linked to the onset of NAFLD. Furthermore, this research indicated that subclinical hypothyroidism prevalence was higher among the patients with NAFLD/NASH than among the healthy controls. Similarly, Loosen et al. compared a cohort of 40,583 patients with NAFLD to an equally sized cohort without NAFLD, and their findings provided firm evidence of the association between NAFLD and subclinical hypothyroidism [35]. However, some studies reported otherwise; for example, Lingad-Sayas et al. discovered that there was no link between subclinical hypothyroidism and NAFLD in a population of 580 adults [66]. Similarly, Ludwig et al. examined 1,276 adults in Germany and determined that neither subclinical nor overt hypothyroidism was linked to NAFLD [67]. The differences in these results may be due to the varying definitions of subclinical hypothyroidism employed by the researchers. For example, Lingad-Sayas et al. characterized subclinical hypothyroidism as having a serum TSH level exceeding 4.5 mIU/L alongside normal FT4 levels [66]. In comparison, Bano et al. set the threshold at a serum TSH level above 4.0 mIU/L with normal FT4 levels [65].

In this research, the examination of an unspecified subgroup (which encompassed studies that did not specify the type of hypothyroidism) showed a notable prevalence of hypothyroidism in NAFLD patients. However, the rate of overt hypothyroidism in NAFLD patients was not significantly elevated. Likewise, a comprehensive study by Lee et al., involving 20,000 participants, did not find a significant link between NAFLD and either subclinical or overt hypothyroidism [68]. Conversely, Bano et al. identified a distinct association between overt hypothyroidism and an increased likelihood of developing NAFLD [65]. The difference in results may be due to variations in the definitions of primary hypothyroidism, including self-reporting by subjects, the need for T4 replacement, abnormal results of thyroid function tests, and different TSH cut-offs used to diagnose hypothyroidism [64,69]. Additionally, ethnicity may play a role in the analysis results, as both studies with adverse outcomes were conducted on Asian subjects. Theoretically, overt hypothyroidism may promote NAFLD development through various factors, including dyslipidemia, obesity, oxidative stress, insulin resistance, and inflammation. Prior research indicates elevated TSH levels can trigger hepatic fat accumulation [70–72]. In hypothyroidism, the decrease in thyroid hormones may lead to an increase in LDL, cholesterol, and triglycerides, as it releases hepatic fatty acids while also lowering HDL, which in turn affects lipid metabolism. As a result, people with evident hypothyroidism often encounter fatty liver infiltration, leading to a heightened risk for NAFLD [62,73].

In this research, NAFLD individuals were shown to have insignificantly lower levels of fT4 and fT3. In contrast with this analysis, Ittermann et al. found a notable link between NAFLD and fT4, but not with fT3, among 3600 adults [74]. Similarly, Ludwig et al., in a cross-sectional study involving 2,445 individuals, discovered a significant inverse correlation between fT4 levels and NAFLD, while no substantial relationship was found for total tT3 or TSH [67]. In contrast, Chung et al. found that increased levels of fT3 were associated with a higher likelihood of NAFLD development [75]. Additionally, Liu et al. conducted a health survey in China involving nearly 2,600 participants, revealing that fT3 was independently associated with NAFLD, whereas T3 did not exhibit a similar association [76]. Intensified fT4 to fT3 conversion is a response to compensate for fat accumulation, aiming to boost energy consumption by enhancing deiodinase activity, which is associated with NAFLD. Theoretically, there must be an association between FT3 levels and NAFLD [77]. The differing results of this study and similar studies could be attributed to the inhibition of T4 to T3 conversion, which is due to uncontrolled variables.

In our analysis, we discovered an elevated level of TSH in individuals with NAFLD. Similarly, studies by Chung et al. and Xu et al. found a positive correlation between NAFLD and TSH levels [51,63]. However, Zhang et al., in a study with over 1,300 participants, revealed that TSH is not considered an independent risk factor for NAFLD development [78]. Likewise, Lee et al. reported no link between NAFLD and hypothyroidism in any of the subtypes among 20,000 Korean study population [68]. The differences in findings between these studies and our current research could stem from the various methods used to categorize participants based on thyroid function or the recruitment strategies employed, whether they were derived from population-based samples or clinical patient groups.

In the current study, age did not show a direct correlation with any of the assessed parameters; however, it may contribute to hypothyroidism development or NAFLD. For instance, Tognini et al. reported a minor influence of older age on hypothyroidism development [79]. Similarly, Zeng et al. analyzed data from 51,407 patients with hypothyroidism and found that older age positively affects the incidence of NAFLD [80]. However, some studies disagreed with the assumed associations between age and NAFLD development [81]. The variation could be related to the differences in the populations studied and potential biases in the sampling process. Ultimately, the female gender was linked with the reciprocal association observed between NAFLD and hypothyroidism and TSH level within NAFLD subjects. These results may stem from different interactions between sex hormones, especially estrogen, liver fat content, and TSH [82].

Also, the likelihood of developing NAFLD is associated with BMI in individuals with hypothyroidism [83]. In this study, hypothyroidism prevalence among NAFLD patients was directly associated with BMI. Demir et al. carried out experiments using rat models with induced hypothyroidism and proposed that hypothyroidism is a causal factor in the onset of NAFLD. They also showed that obesity could mediate the significant link between hypothyroidism and NAFLD in these rat subjects [84]. Zhang et al. examined the association between components of metabolic syndrome and serum thyrotropin levels. Their results indicated that BMI was notably higher in adolescents with subclinical hypothyroidism compared to healthy controls [85].

In addition to epidemiologic rigor, biological explanations are necessary to strengthen the bidirectional link between NAFLD and subclinical – but not overt – hypothyroidism. Since no single underlying mechanism can fully explain the NAFLD development in hypothyroid patients, a combination of pathways is likely responsible for this association. Thyroid hormones, especially T3, regulate hepatic lipid metabolism, and their signaling pathways often influence genes involved in β-oxidation, lipogenesis, and lipoprotein metabolism [86]. Dysregulation of T3 and TSH secretion in subclinical hypothyroidism leads to decreased intrahepatic lipid breakdown, impaired VLDL secretion, and secondary dyslipidemia (characterized by increased serum cholesterol and LDL-C levels) [78]. Research conducted both in vitro and in vivo has demonstrated that the action of thyrotropin, via its receptors, promotes preadipocyte-to-adipocyte conversion, mediated by cAMP-dependent protein kinases [85]. Additionally, subclinical hypothyroidism is consistently associated with insulin resistance (and consequently de novo lipogenesis), a primary driver for NAFLD [57,87].

**Fig 5:**
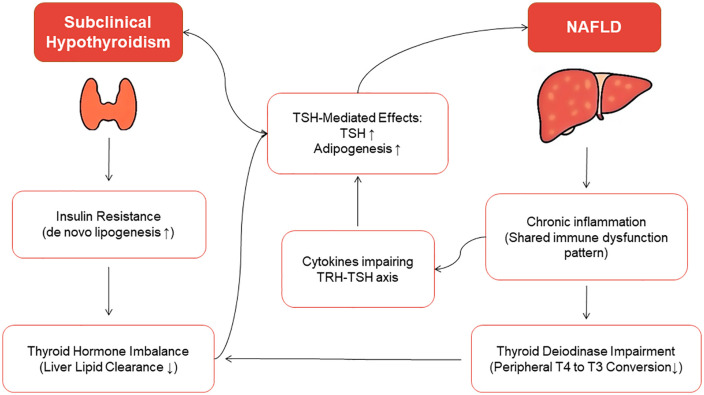
Diagram summarizing the interplay between NAFLD and SCH, highlighting mechanisms such as dyslipidemia, insulin resistance, and inflammation.

Some of the mechanisms mentioned earlier are also found in overt hypothyroidism, but interestingly, there is no observed link between overt hypothyroidism and NAFLD. While no single mechanism can fully explain this, some clues can be offered. First, subclinical hypothyroidism, which is marked by ongoing low-grade distress, may trigger the initiation and progression of NAFLD, a disease that itself develops gradually and progresses slowly [64,88]. Second, in overt hypothyroidism, a wide range of adaptations may be observed, such as reduced lipid flux into the liver, and these may differ from those in subclinical hypothyroidism [88]. At last, thyroid functional tests may detect “Sick Euthyroid” confounders – having low levels of thyroid hormones due to other systemic illnesses and not hypothyroidism – as overt hypothyroidism patients [89]. In the other direction, some mechanisms can explain how NAFLD can connect with subclinical hypothyroidism. As NAFLD is characterized by chronic low-grade inflammation, featuring several mediators such as IL-6 or TNF-α, which can lead to subtle changes in TSH or Thyrotropin-Releasing Hormone (TRH) secretion, or share a similar pattern with several thyroid autoimmune diseases, like Hashimoto’s thyroiditis, which may be linked to subclinical hypothyroidism [90,91]. NAFLD may also induce slight changes in the activity of thyroid deiodinase enzymes, which are crucial in the metabolism of T3 and T4, leading to a decrease in their activity, via inflammatory factors [92]. The mechanisms linking subclinical hypothyroidism and NAFLD are summarized in Fig 5.

More recently, molecular studies have shown that activating thyroid hormone receptor-β (TRβ), for instance, with selective agonists like resmetirom, boosts liver fat oxidation and decreases its buildup, even without changes in body weight [93,94]. At the transcriptional level, TRβ regulates both fat synthesis and breakdown via the glucose-responsive transcription factor ChREBP [95]. When T3 binds to TRβ1, it increases ChREBP activation and its DNA-binding affinity. This drives the expression of genes involved in fat synthesis while also promoting fat oxidation pathways [95]. In addition, thyroid hormone signaling encourages lipophagy and autophagy [96]. This helps move lipid droplets via autophagic processes, such as ATG5, allowing freed fatty acids to enter mitochondrial fatty acid oxidation [96]. On the other hand, a lack of TRβ or its dysfunction, such as in models with dominant-negative Thrb mutations, results in decreased fat oxidation and improper activation of PPAR-γ. This leads to fat buildup and steatosis [93]. Taken altogether, thyroid dysfunction can worsen insulin resistance by altering adipokine levels, with hypothyroid patients often having high leptin and low adiponectin, impairing liver insulin signaling and fat buildup. This supports that reduced thyroid hormone signaling or TRβ dysfunction may impair hepatic fat oxidation, disrupt lipid balance, and worsen insulin resistance-related NAFLD.

He et al. showed that NAFLD and hypothyroidism (both overt and subclinical) were correlated (OR=1.72) [97]. In 2018, Mantovani et al. performed a meta-analysis of 12 articles and calculated an OR = 1.42 for prevalent NAFLD incidence among hypothyroid patients [64]. Then Zeng et al. showed that NAFLD incidence was more prevalent in hypothyroid patients, and risk factors for this interplay were identified; age, BMI, and TSH were found to increase NAFLD presence in hypothyroid patients [83]. All previous analyses showed a high between-study heterogeneity [83,97,64]. Finally, in 2024, Xiang et al. showed that high fT3, unlike fT4 and TSH, was correlated with the incidence of NAFLD [98]. Our study, unlike previous studies, conducted a meta-analysis for each direction of the interplay between NAFLD and hypothyroidism, featuring a higher number of articles and participants (35 studies and 38,877,762 participants), suggesting that the two diseases are associated bidirectionally. Additionally, TSH, unlike fT3 and fT4, was significantly higher in patients with NAFLD. Ultimately, several subgroup analyses and meta-regression analyses revealed that age and BMI, unlike region and study design in some instances, did not substantially enhance outcomes. The NAFLD diagnostic method was also not fully responsible for the calculated heterogeneity, which was a significant limitation of this study.

High heterogeneity and the existing bias among the included studies suggest a potential overrepresentation of studies with significant results in the literature, which could exaggerate overall effect estimates, compromise the reliability of conclusions, and reduce their robustness. Several factors may have contributed to this heterogeneity. Firstly, the methods used to diagnose NAFLD varied across studies, including ultrasound (US), liver biopsy, and transient elastography. Compared to liver biopsy, most imaging modalities used in the included studies have lower sensitivity and accuracy for detecting steatohepatitis [99]. Secondly, definitions of primary hypothyroidism differed among studies, ranging from self-reported cases with levothyroxine treatment to laboratory-confirmed thyroid function tests, with variable TSH and free T4 cutoffs for subclinical and overt hypothyroidism. Data based on self-reported hypothyroidism or levothyroxine use require cautious interpretation, as many patients may be euthyroid or have subclinical hypothyroidism based on mean serum TSH levels. Limited monitoring of thyroid hormones and antibodies, along with incomplete information on hypothyroidism duration, further restricted the ability to evaluate the association between hypothyroidism severity and NAFLD. To account for these variations, two dedicated columns in Table 1 summarize study definitions, and both meta-regression and subgroup analyses were performed; however, these analyses did not fully explain the observed heterogeneity. Thirdly, a major limitation of this study is the overwhelming female representation in the pooled sample (99%), primarily driven by the large female cohort in the study by Boustany et al. A significant association between hypothyroidism and NAFLD remained even after removing this study (S4 Table), yet the gender imbalance limits the generalizability of the findings to male populations. Notably, females are more prone to thyroid dysfunction due to factors such as immune function [100]. Fourthly, other between-study differences—such as region, ethnicity, and observational study design—may have contributed to heterogeneity. Subgroup analyses were therefore conducted based on country and study design. Finally, despite the aforementioned attempts to reduce heterogeneity, it remained substantial, and Egger’s test and funnel plots suggested potential publication bias for some outcomes, particularly TSH levels, possibly reflecting underreporting of studies with null results and thereby inflating effect estimates, underscoring the need for cautious interpretation of the pooled results.

## Conclusion

This study indicates a significant association between NAFLD and subclinical or unspecified hypothyroidism. However, overt hypothyroidism was not significantly associated with NAFLD risk. Additionally, the incidence of overall and subclinical hypothyroidism was significantly higher among patients with NAFLD, whereas overt hypothyroidism showed no substantial link. Despite these findings, future studies should adjust for confounding factors, include diverse ethnic groups, involve balanced gender representation, and assess hypothyroidism duration and severity to better clarify this relationship. Although subgroup and meta-regression analyses were performed, I² values remained high for most outcomes, influencing the interpretation and robustness of the pooled results.

## Supporting information

S1 Fig Funnel plot describing the association between hypothyroidism subtypes and the risk of NAFLD or NASH. (TIF)

S2 Fig Funnel plot describing the association between hypothyroidism and the risk of NAFLD vs NASH. (TIF)

S3 Fig The association between hypothyroidism and the risk of NAFLD or NASH based on sub-group analysis (division into groups based on their study design). (TIF)

S4 Fig The association between hypothyroidism and the risk of NAFLD or NASH based on sub-group analysis (division into groups based on their study location). (TIF)

S5 Fig The association between hypothyroidism and the risk of NAFLD or NASH based on sub-group analysis (division into groups based on their NAFLD/NASH detection method). (TIF)

S6 Fig Funnel plot describing the association between NAFLD or NASH and the risk of hypothyroidism. (TIF)

S7 Fig The association between NAFLD or NASH and the risk of hypothyroidism based on subgroup analysis (division into groups based on their study design). (TIF)

S8 Fig Forest plot describing the association between NAFLD or NASH and the risk of hypothyroidism based on subgroup analysis (division into groups based on their study design). (TIF)

S9 Fig Forest plot describing the association between NAFLD or NASH and the risk of hypothyroidism based on subgroup analysis (division into groups based on their NAFLD/NASH detection method). (TIF)

S10 Fig Forest and funnel plot describing the association between NAFLD and fT3 serum level. (TIF)

S11 Fig Forest and funnel plot describing the association between NAFLD and fT4 serum level. (TIF)

S12 Fig Forest plot describing the association between NAFLD and fT4 serum level after sensitivity analysis and removal of Kassem’s study. (TIF)

S13 Fig Forest plot describing the association between NAFLD and fT3 serum level based on subgroup analysis (division into groups based on their NAFLD/NASH detection method). (TIF)

S14 Fig Forest plot describing the association between NAFLD and fT4 serum level based on subgroup analysis (division into groups based on their NAFLD/NASH detection method). (TIF)

S15 Fig Forest and Funnel plot assessing the association between the TSH level and NAFLD (prior to sensitivity analysis). (TIF)

S16 Fig Forest plot describing the association between NAFLD and TSH serum level based on subgroup analysis (division into groups based on their NAFLD/NASH detection method). (TIF)

S1 Table Cross-sectional studies bias assessment. (DOCX)

S2 Table Cohort studies bias assessment. (DOCX)

S3 Table Case-control studies bias assessment. (DOCX)

S4 Table Sensitivity analysis for the risk of Hypothyroidism in the context of NAFLD. (DOCX)

S5 Table Sensitivity analysis for the risk of NAFLD in the context of hypothyroidism. (DOCX)

S6 Table Sensitivity analysis for the fT3 level in the context of NAFLD. (DOCX)

S7 Table Sensitivity analysis for the fT4 level in the context of NAFLD. (DOCX)

S8 Table Sensitivity analysis for the TSH level in the context of NAFLD. (DOCX)

S1 File PRISMA checklist. (DOCX)

S2 File Analysis datasheet. (XLSX)
