# Supplementary figures and images for: The reciprocal relationship between non-alcoholic fatty liver disease and hypothyroidism: A systematic review and meta-analysis of about 39 million individuals
Source: PLoS One. 2025 Dec 18;20(12):e0338413. doi: 10.1371/journal.pone.0338413 (PMC12714247; doi:10.1371/journal.pone.0338413)

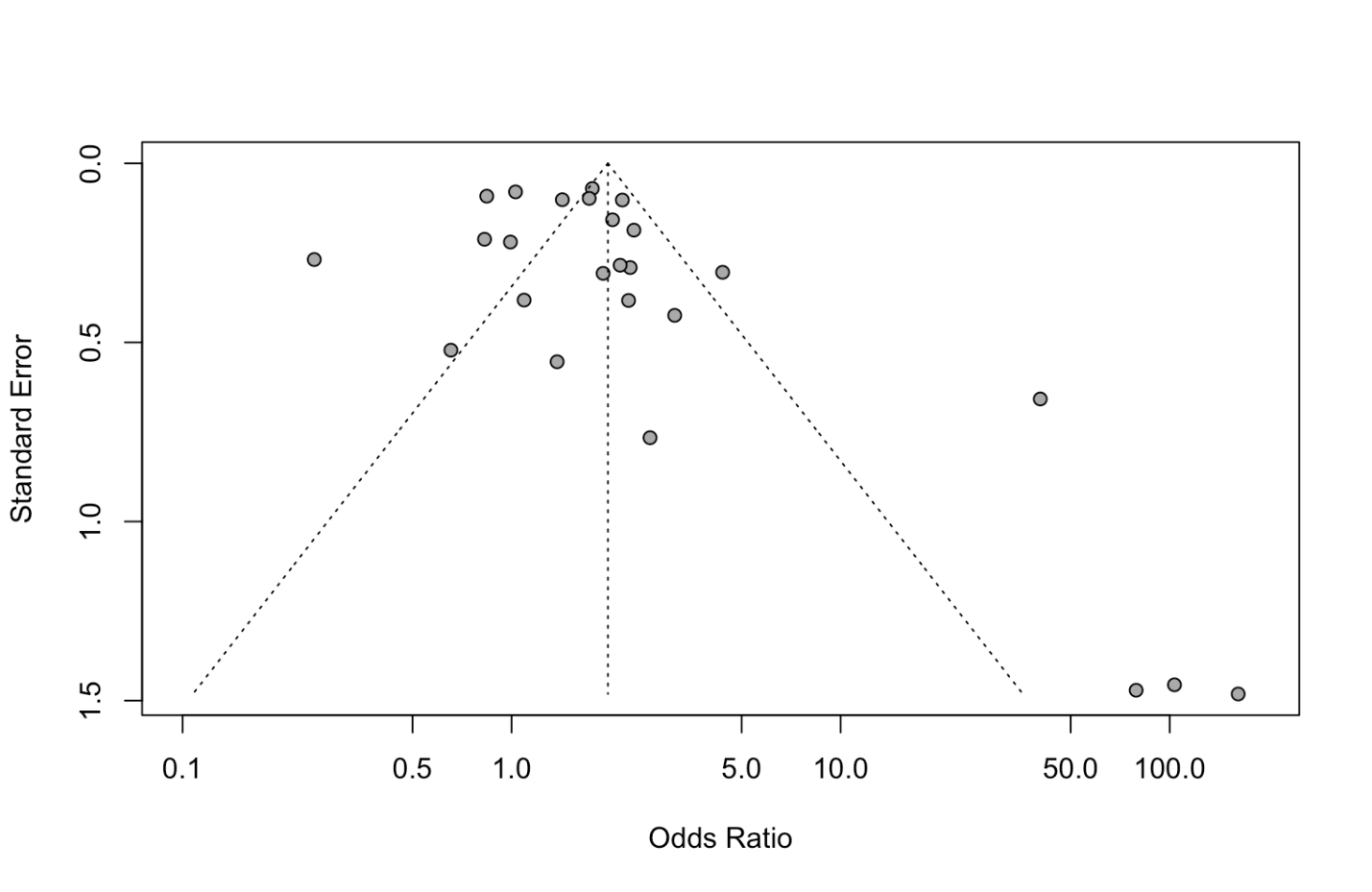

Supplement: S1 Fig — (TIF) [file pone.0338413.s001.tif]

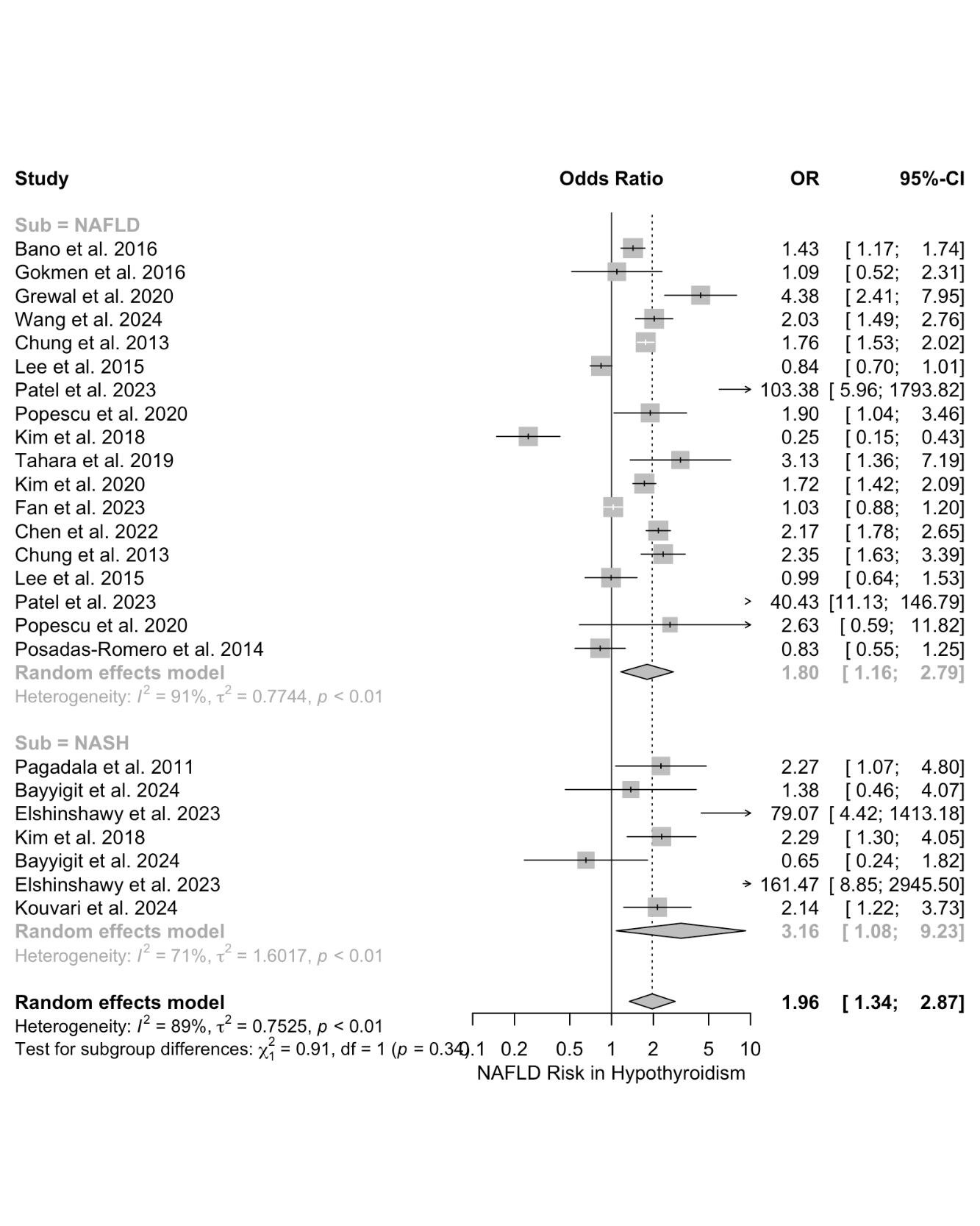

Supplement: S2 Fig — (TIF) [file pone.0338413.s002.tif]

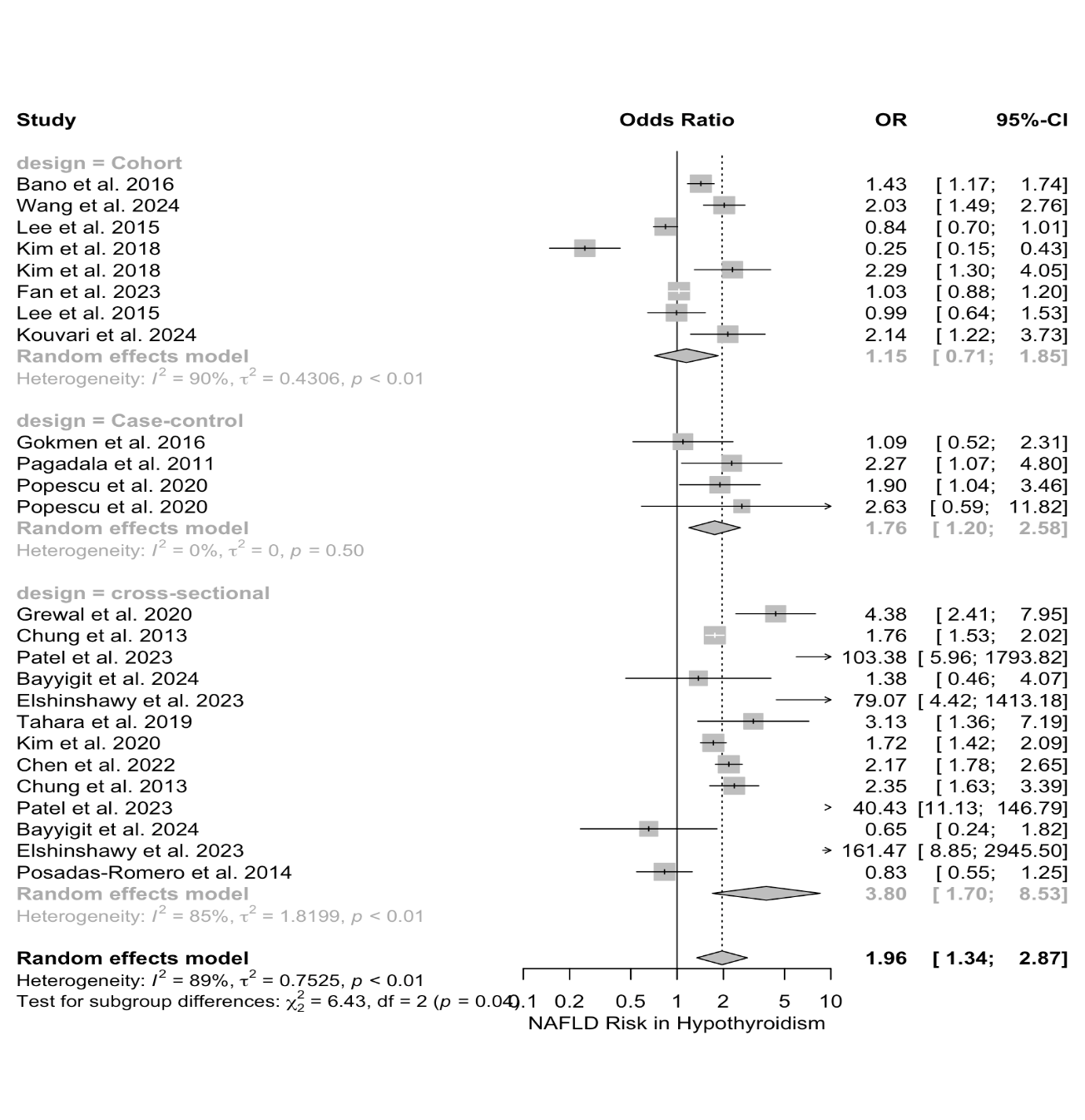

Supplement: S3 Fig — (TIF) [file pone.0338413.s003.tif]

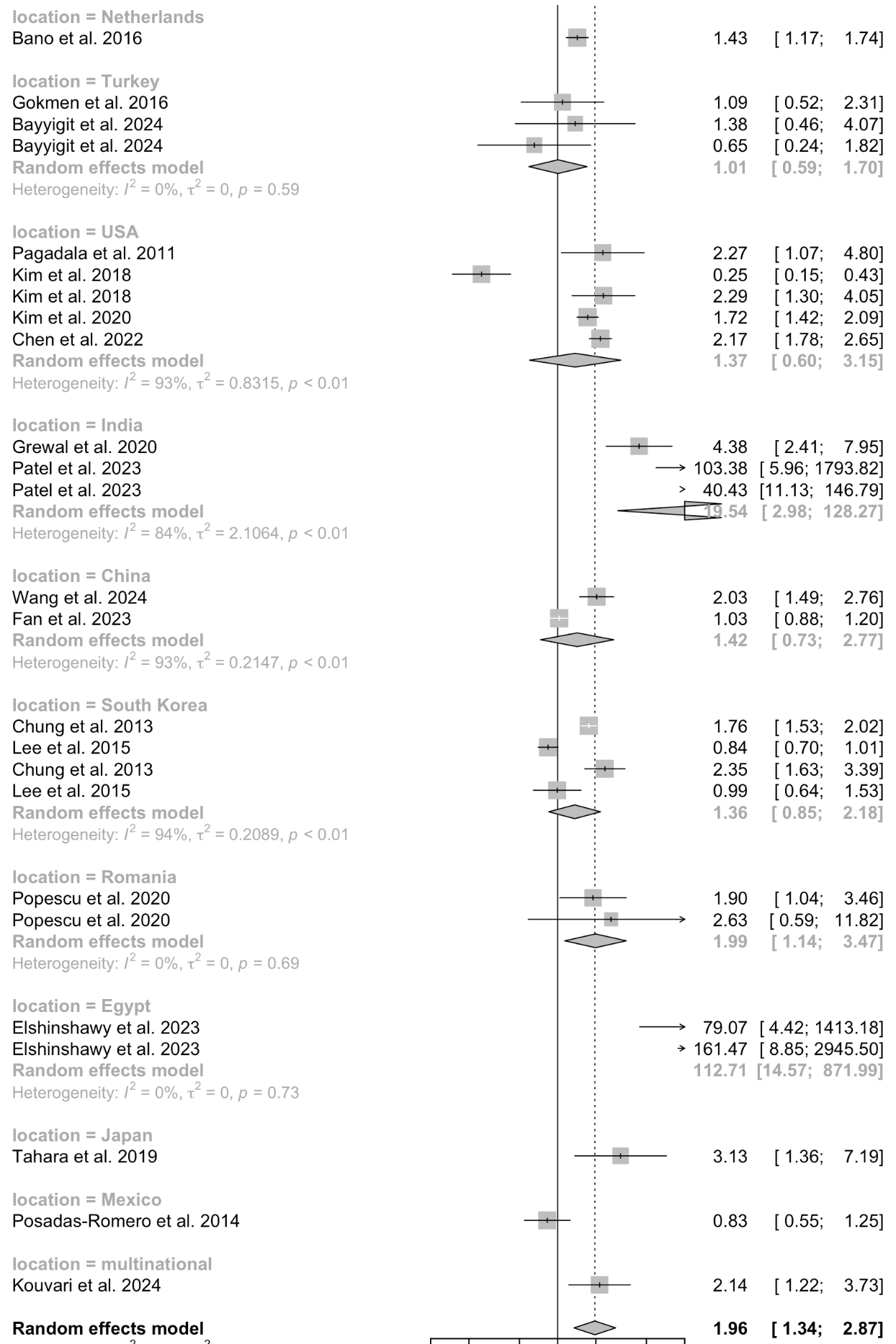

Supplement: S4 Fig — (TIF) [file pone.0338413.s004.tif]

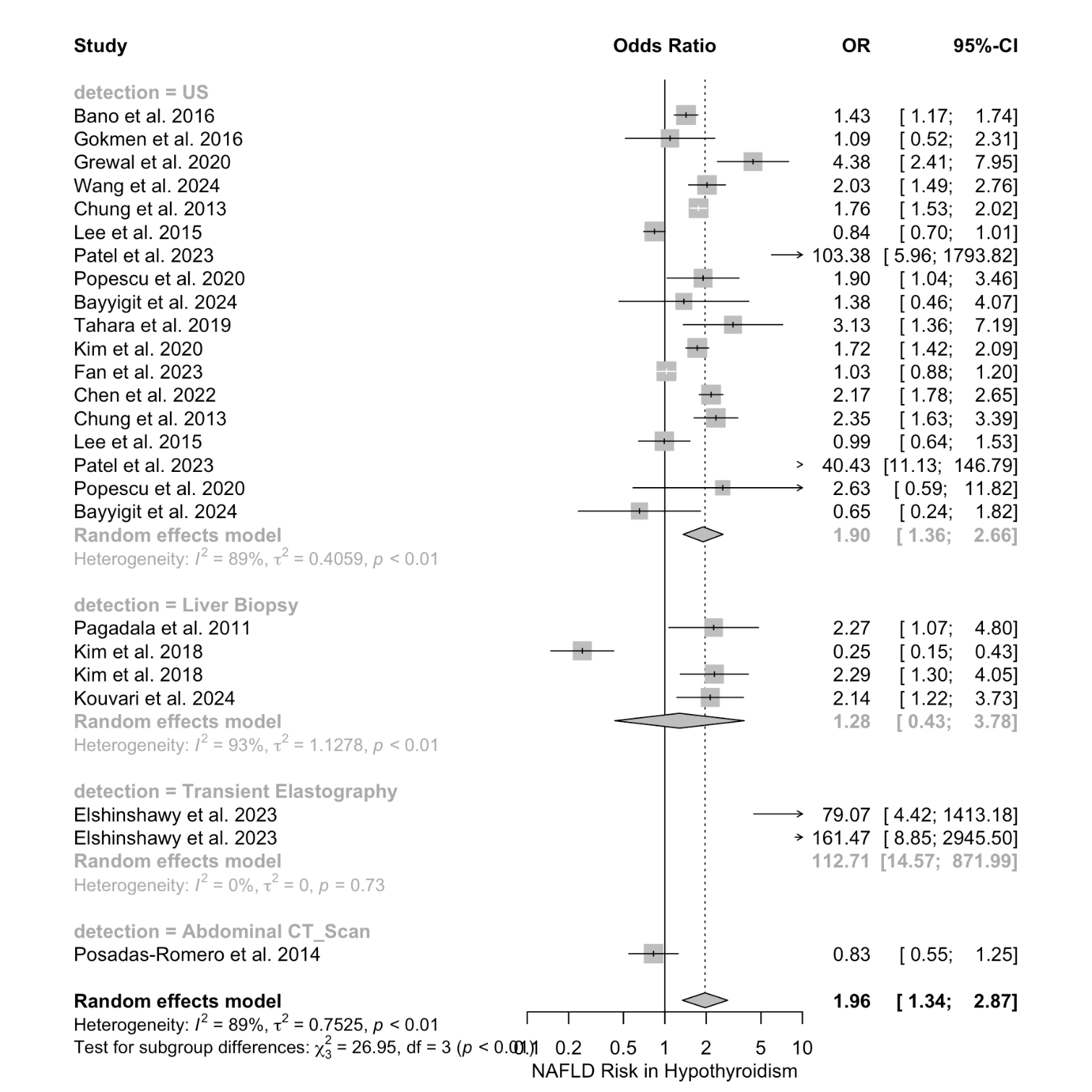

Supplement: S5 Fig — (TIF) [file pone.0338413.s005.tif]

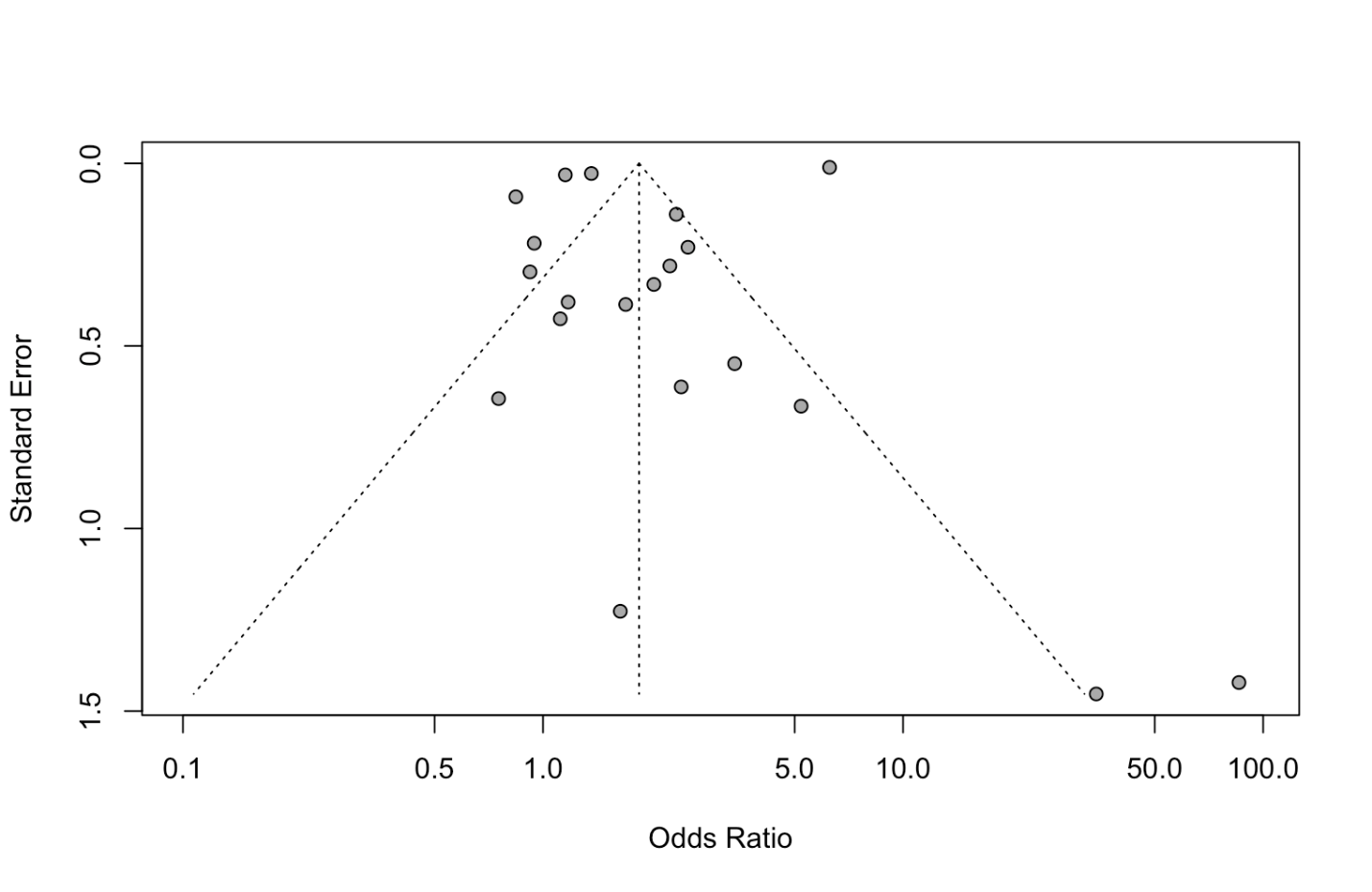

Supplement: S6 Fig — (TIF) [file pone.0338413.s006.tif]

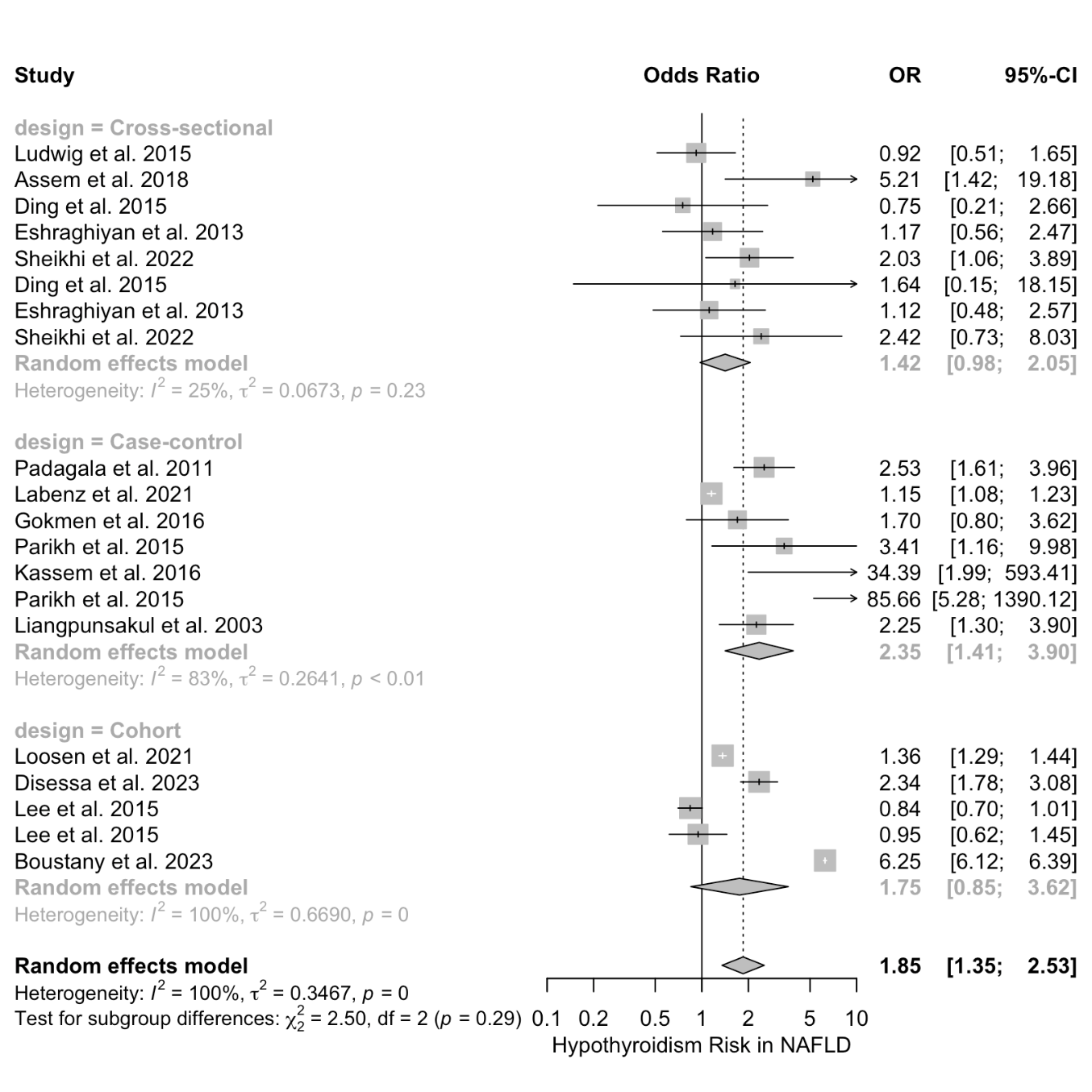

Supplement: S7 Fig — (TIF) [file pone.0338413.s007.tif]

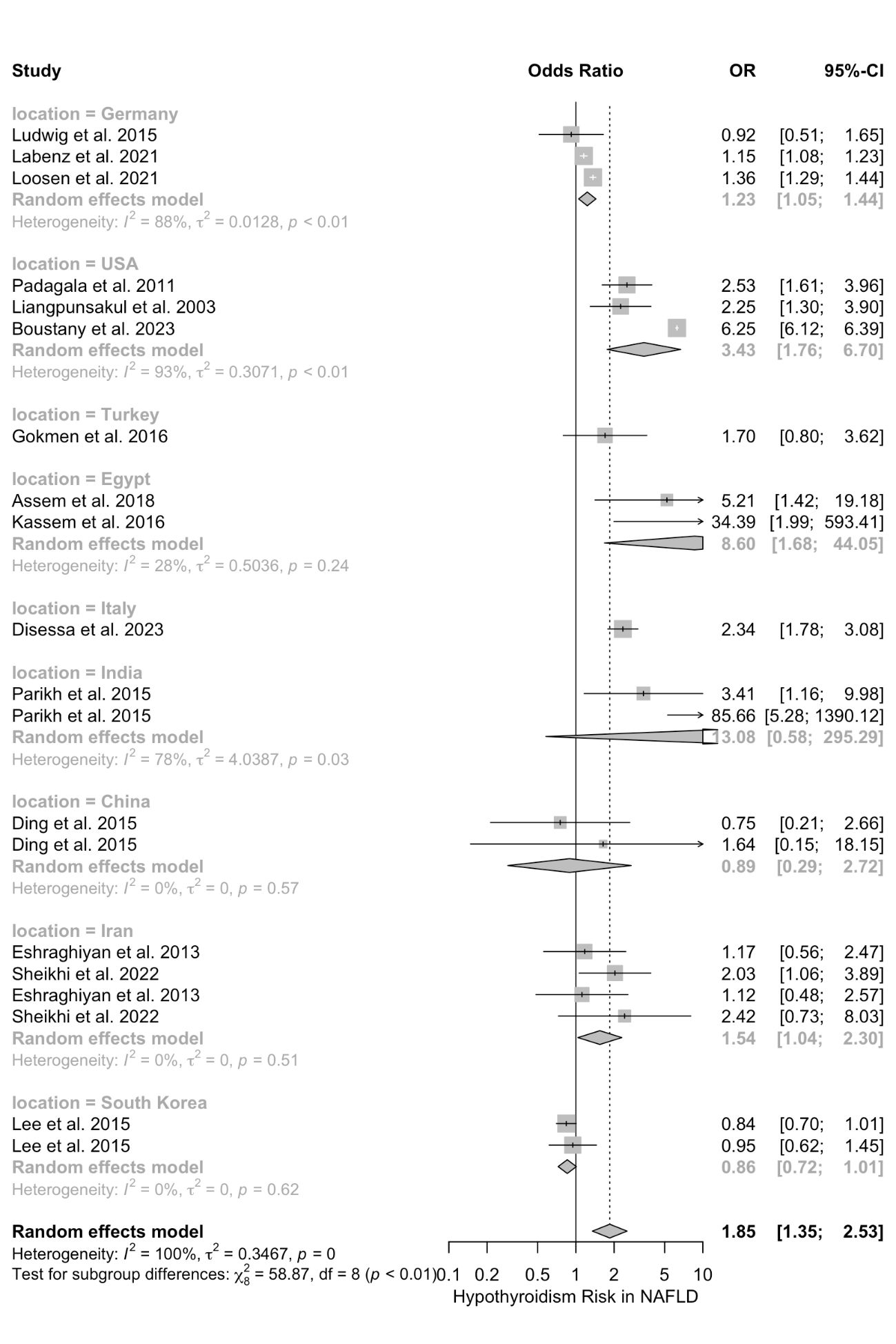

Supplement: S8 Fig — (TIF) [file pone.0338413.s008.tif]

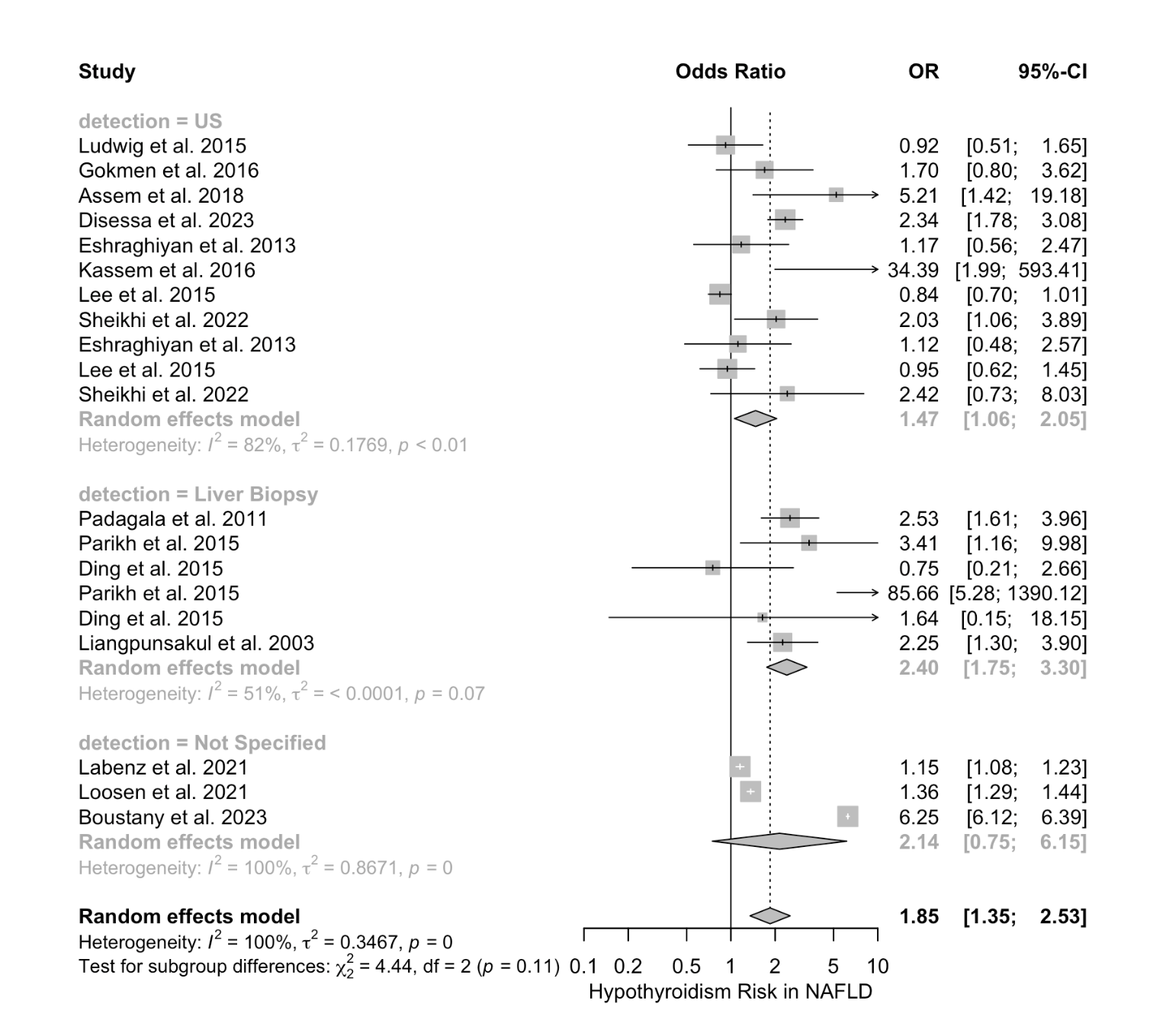

Supplement: S9 Fig — (TIF) [file pone.0338413.s009.tif]

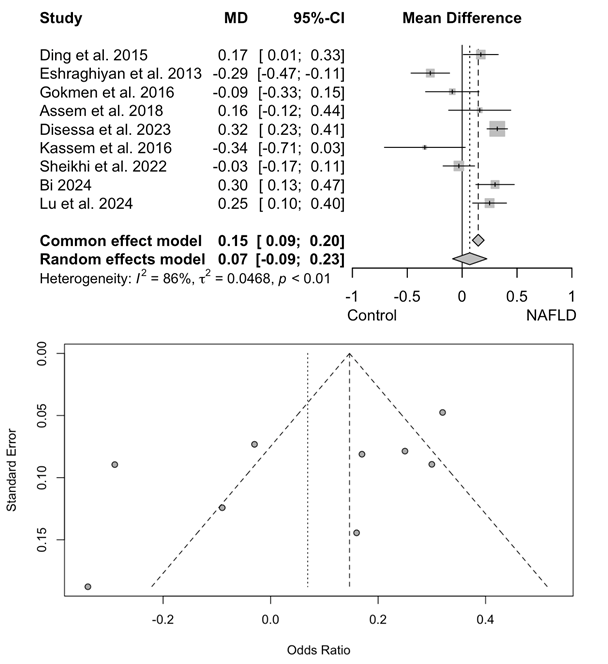

Supplement: S10 Fig — (TIF) [file pone.0338413.s010.tif]

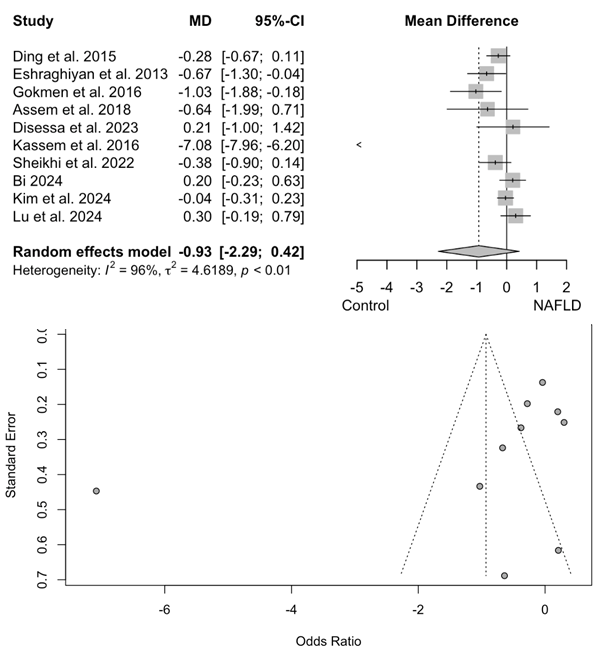

Supplement: S11 Fig — (TIF) [file pone.0338413.s011.tif]

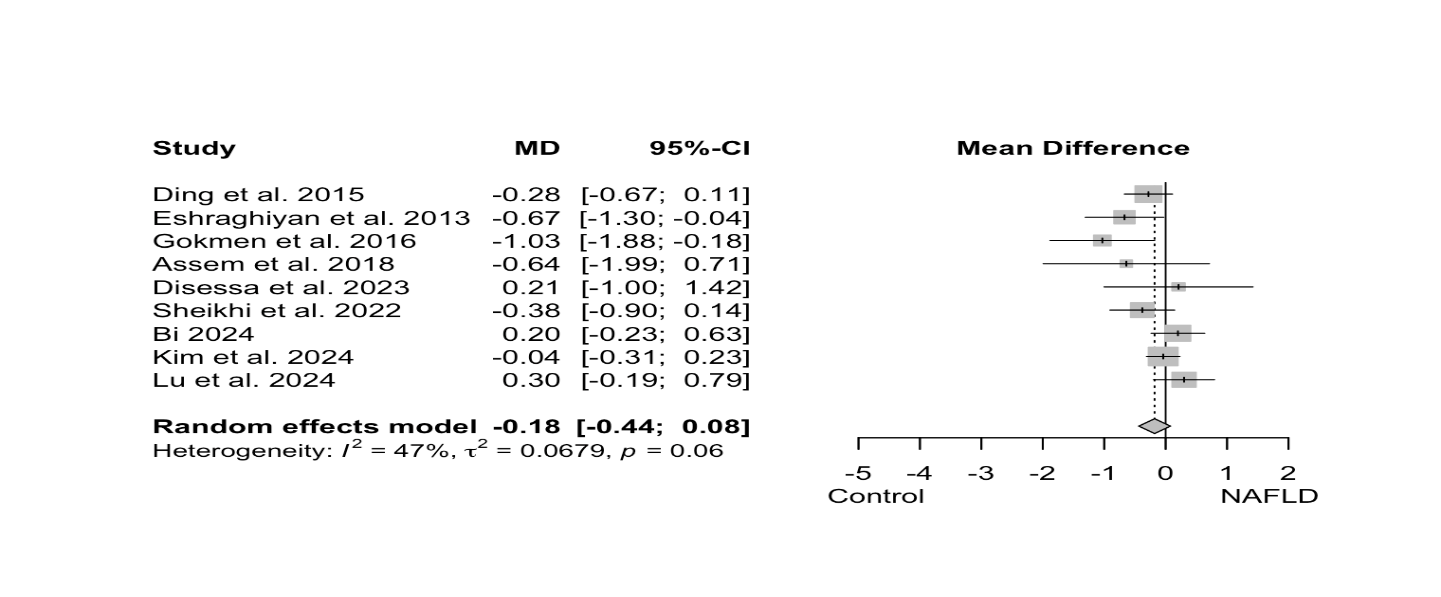

Supplement: S12 Fig — (TIF) [file pone.0338413.s012.tif]

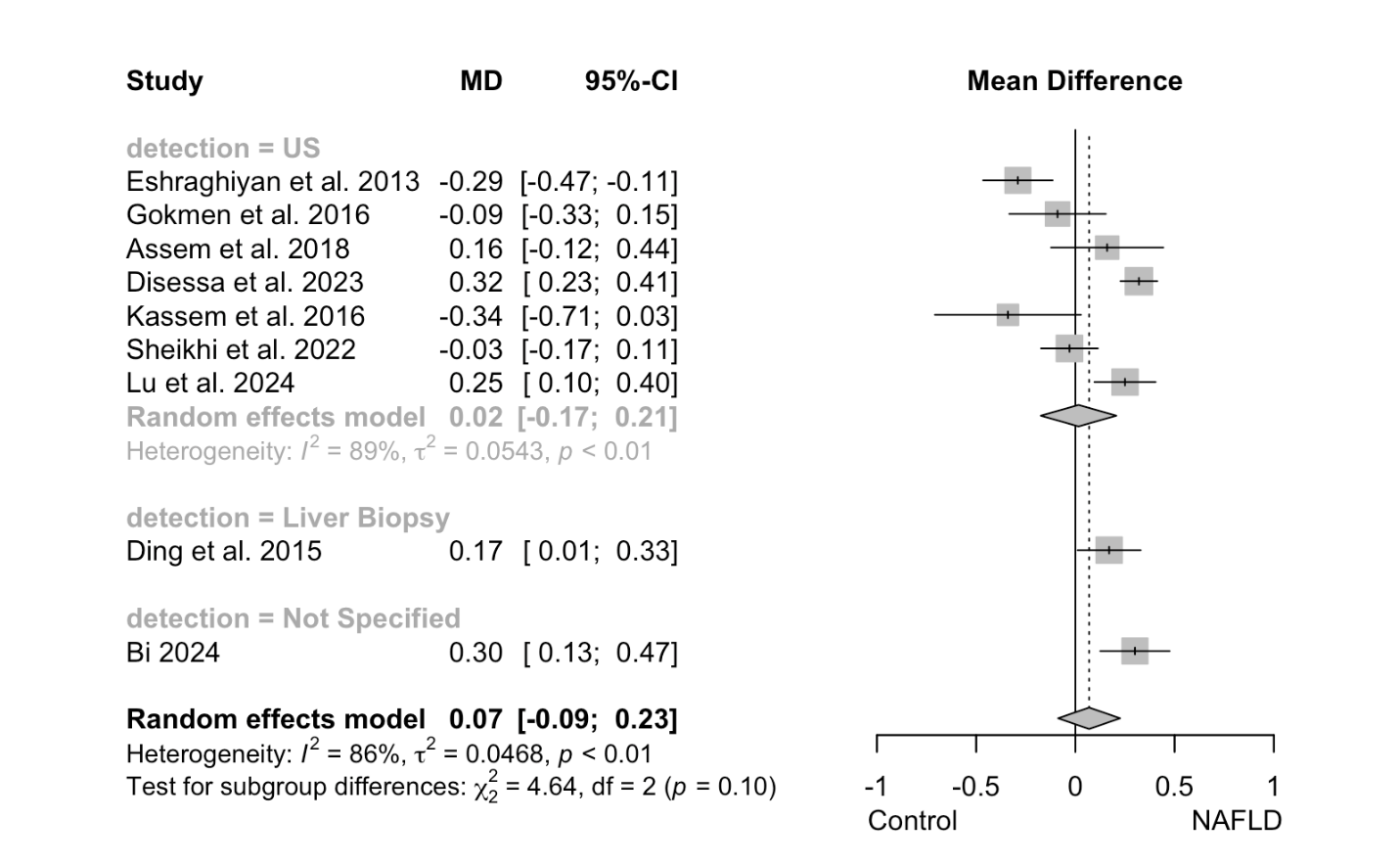

Supplement: S13 Fig — (TIF) [file pone.0338413.s013.tif]

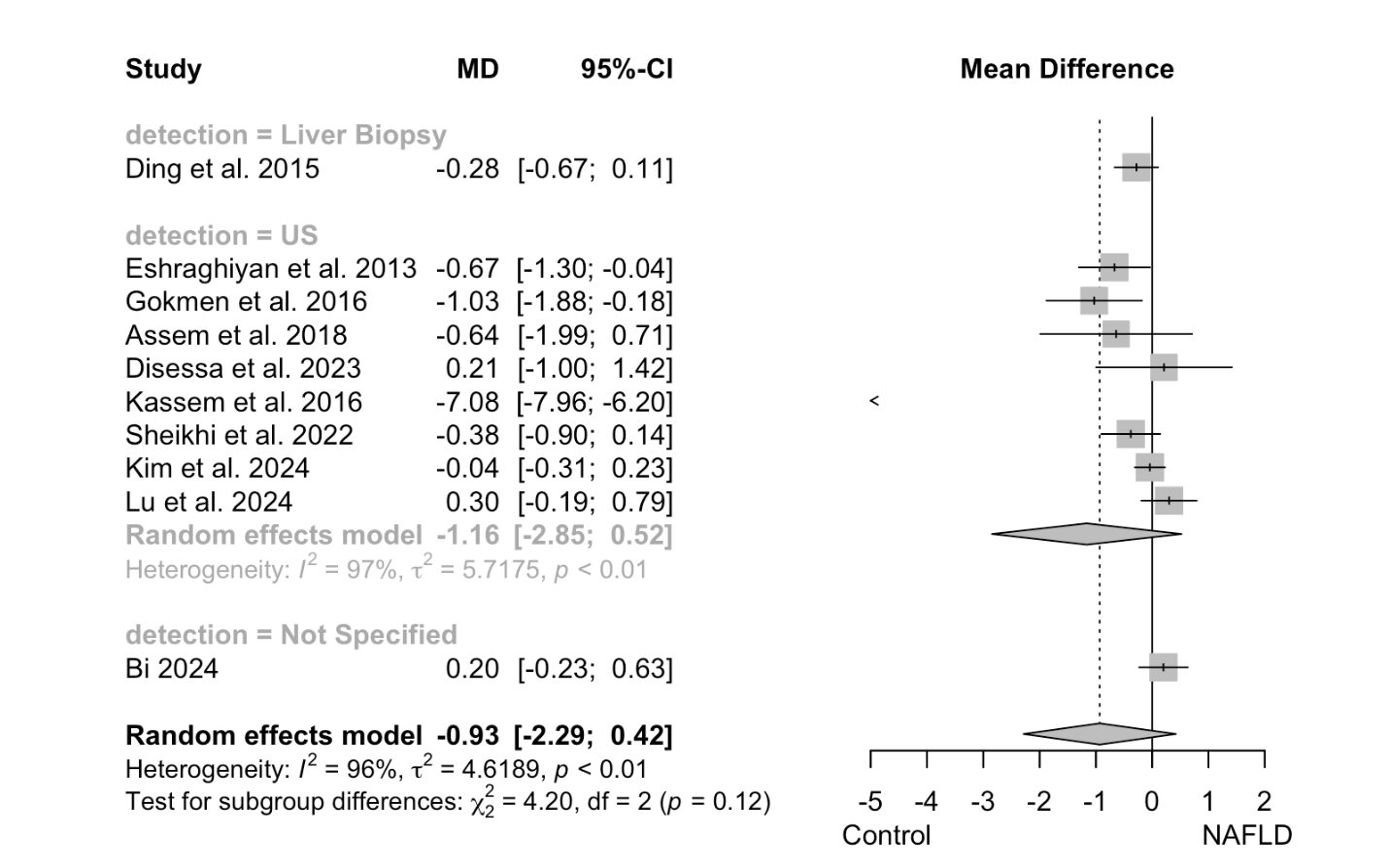

Supplement: S14 Fig — (TIF) [file pone.0338413.s014.tif]

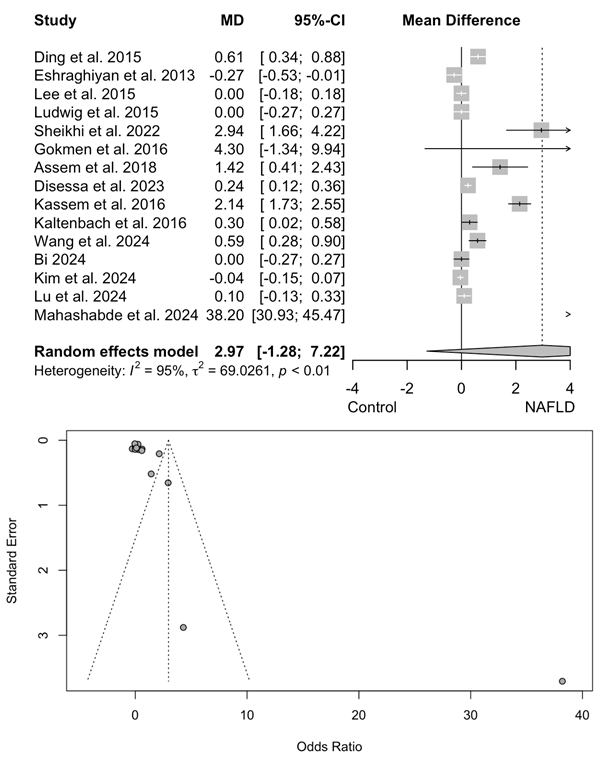

Supplement: S15 Fig — (TIF) [file pone.0338413.s015.tif]

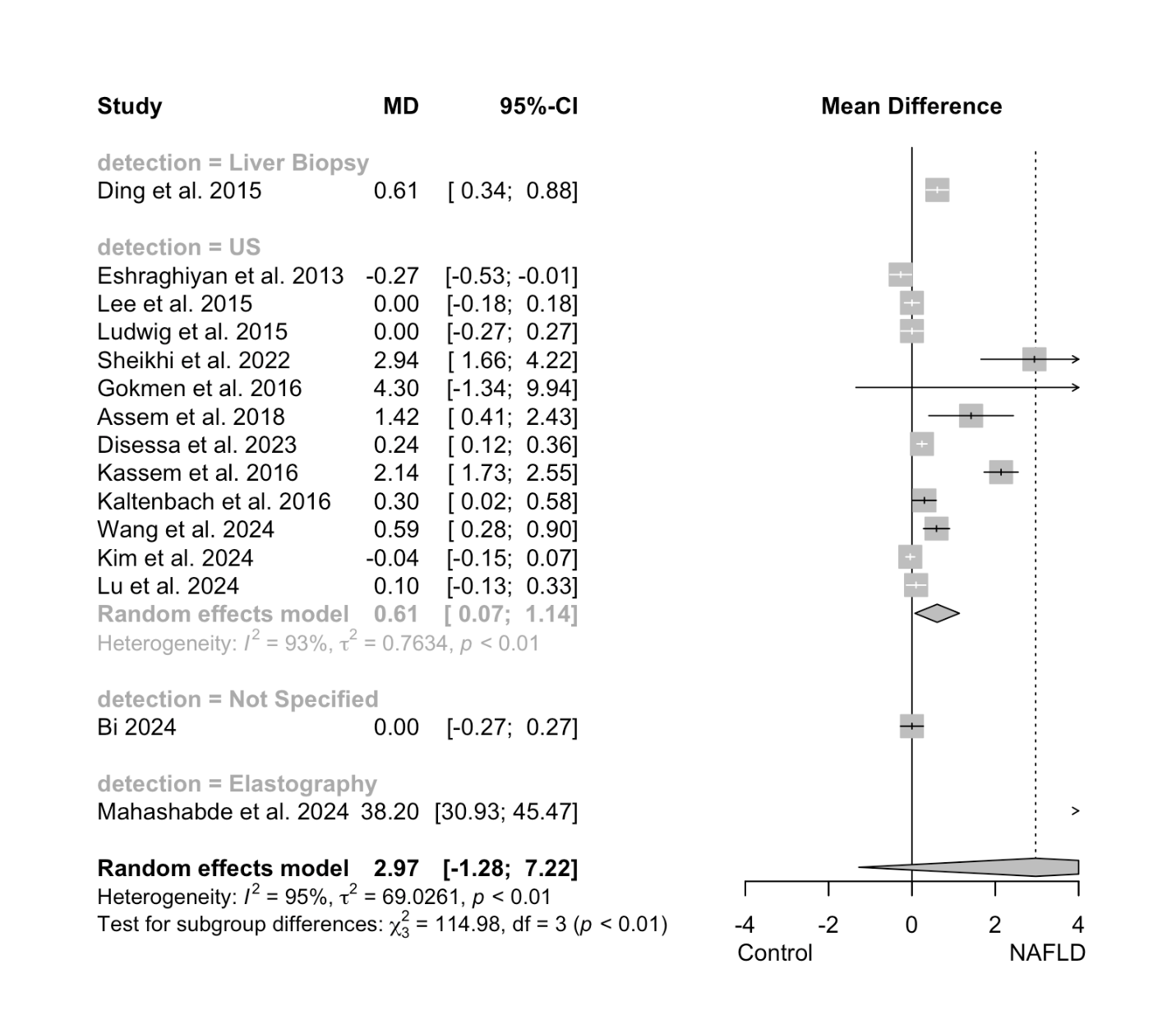

Supplement: S16 Fig — (TIF) [file pone.0338413.s016.tif]
