# Supplementary material for: The reciprocal relationship between non-alcoholic fatty liver disease and hypothyroidism: A systematic review and meta-analysis of about 39 million individuals
Source: PLoS One. 2025 Dec 18;20(12):e0338413. doi: 10.1371/journal.pone.0338413 (PMC12714247; doi:10.1371/journal.pone.0338413)
Supplement: S1 Table — (DOCX) [file pone.0338413.s017.docx]

| Author | 1 | 2 | 3 | 4 | 5 | 6 | 7 | 8 |
| --- | --- | --- | --- | --- | --- | --- | --- | --- |
| **Bayyigit et al. 2024 [1]** | Yes | Yes | Yes | Yes | Yes | No | Unclear | Yes |
| **Bi et al. 2024 [2]** | Yes | Yes | Unclear | Yes | Yes | Yes | Yes | Yes |
| **Kouvari et al. 2024 [3]** | Yes | Yes | Yes | Yes | Yes | Yes | Yes | Yes |
| **Mahashabde et al. 2024 [4]** | Yes | Yes | No | Yes | Yes | No | Yes | Unclear |
| **Elshinshawy et al. 2023 [5]** | Yes | No | Yes | Yes | Yes | Yes | No | Yes |
| **Patel et al. 2023 [6]** | Yes | Yes | Yes | Yes | Yes | Unclear | Unclear | Yes |
| **Chen et al. 2022 [7]** | Yes | No | Yes | Yes | Yes | Yes | No | Yes |
| **Sheikhi et al. 2022 [8]** | Yes | Yes | Yes | Yes | Unclear | Yes | No | Yes |
| **Grewal et al. 2020 [9]** | Yes | Yes | No | Yes | Yes | Unclear | Unclear | Yes |
| **Tahara et al. 2019 [10]** | Yes | Yes | Yes | Yes | Yes | Yes | Unclear | Yes |
| **Assem et al. 2018 [11]** | Yes | Yes | Unclear | Yes | No | No | Yes | Yes |
| **Kaltenbach et al. 2016 [12]** | Yes | Yes | Yes | No | Yes | Yes | No | Yes |
| **Ding et al. 2015 [13]** | Yes | Yes | Yes | Unclear | Yes | Yes | Unclear | Yes |
| **Ludwig et al. 2015 [14]** | Yes | Yes | Unclear | Yes | Yes | Yes | Unclear | Yes |
| **Posadas-Romero et al. 2014 [15]** | Yes | Yes | No | Yes | Yes | Yes | Unclear | Yes |
| **Chung et al. 2013 [16]** | Yes | Yes | Yes | Unclear | Yes | Yes | No | Yes |
| **Eshraghian et al. 2013 [17]** | Yes | Yes | No | Yes | Yes | Yes | Unclear | Yes |

| Author | 1 | 2 | 3 | 4 | 5 | 6 | 7 | 8 | 9 | 10 | 11 |
| --- | --- | --- | --- | --- | --- | --- | --- | --- | --- | --- | --- |
| **Kim et al. 2024 [18]** | Yes | Yes | Yes | Yes | Yes | Yes | No | Yes | Yes | No | Yes |
| **Lu et al. 2024 [19]** | Unclear | Yes | Yes | Yes | Yes | Yes | Yes | No | No | No | Yes |
| **Wang et al. 2024 [20]** | Yes | Yes | Yes | Yes | Yes | Yes | Yes | Yes | No | NA | Yes |
| **Boustany et al. 2023 [21]** | Yes | Yes | Yes | Yes | Yes | Yes | Yes | NA | NA | NA | Yes |
| **Di Sessa et al. 2023 [22]** | Yes | Yes | Yes | Yes | Yes | Unclear | Yes | NA | NA | NA | Yes |
| **Fan et al. 2023 [23]** | Yes | Yes | Yes | Yes | Yes | Unclear | Yes | No | NA | NA | Yes |
| **Loosen et al. 2021 [24]** | Yes | Yes | Unclear | Yes | Yes | Yes | Unclear | Yes | Unclear | Unclear | Yes |
| **Kim et al. 2020 [25]** | Yes | Yes | Yes | Yes | Yes | No | Yes | Yes | No | Yes | Yes |
| **Kim et al. 2018 [26]** | Yes | Yes | Yes | Yes | Yes | No | Yes | No | Unclear | Unclear | Yes |
| **Bano et al. 2016 [27]** | Yes | Yes | Yes | Yes | Yes | Unclear | Unclear | Yes | Unclear | NA | Yes |
| **Lee et al. 2015 [28]** | Yes | Yes | Yes | Yes | Yes | Yes | Unclear | Yes | No | NA | Yes |

| Author | 1 | 2 | 3 | 4 | 5 | 6 | 7 | 8 | 9 | 10 |
| --- | --- | --- | --- | --- | --- | --- | --- | --- | --- | --- |
| **Labenz et al 2021 [29]** | Yes | Yes | Yes | Yes | Yes | Yes | Yes | Yes | Yes | Yes |
| **Popescu et al. 2020 [30]** | Yes | No | Yes | Yes | Yes | Yes | No | Yes | No | No |
| **Gokmen et al. 2016 [31]** | Yes | Unclear | Unclear | Yes | Yes | Yes | Yes | Yes | Unclear | Yes |
| **Kassem et al. 2016 [32]** | Yes | No | No | Yes | Yes | Yes | Yes | Yes | Unclear | Unclear |
| **Parikh et al. 2015 [33]** | No | Unclear | Yes | Unclear | Yes | Yes | Yes | Yes | Unclear | Yes |
| **Pagadala et al. 2011 [34]** | Yes | Yes | Yes | Unclear | Yes | Yes | Yes | Yes | Unclear | Yes |
| **Liangpunsakul et al. 2003 [35]** | Yes | Yes | Yes | Unclear | Unclear | Yes | Yes | Yes | Unclear | Yes |

| **Study** | **Estimate** | **CI_lb** | **CI_ub** | **p_value** | **Tau2** | **I2** |
| --- | --- | --- | --- | --- | --- | --- |
| **Liangpunsakul et al. 2003** | 1.833019 | 1.313228 | 2.558549 | 0.000369 | 0.372176 | 98.88072 |
| **Padagala et al. 2011** | 1.81556 | 1.302217 | 2.531267 | 0.000436 | 0.367091 | 98.86275 |
| **Eshraghiyan et al. 2013.1** | 1.901517 | 1.367499 | 2.644074 | 0.000133 | 0.365315 | 98.86237 |
| **Eshraghiyan et al. 2013.2** | 1.902246 | 1.370049 | 2.641176 | 0.000123 | 0.362923 | 98.85555 |
| **Ludwig et al. 2015** | 1.931768 | 1.396501 | 2.672199 | 6.97E-05 | 0.347892 | 98.80413 |
| **Parikh et al. 2015.1** | 1.768455 | 1.302439 | 2.401214 | 0.000259 | 0.319306 | 98.70366 |
| **Parikh et al. 2015.2** | 1.803598 | 1.306584 | 2.489671 | 0.000336 | 0.351115 | 98.81848 |
| **Lee et al. 2015.1** | 1.959424 | 1.42685 | 2.690781 | 3.23E-05 | 0.322052 | 98.65574 |
| **Lee et al. 2015.2** | 1.936944 | 1.399573 | 2.680639 | 6.67E-05 | 0.346339 | 98.79455 |
| **Ding et al. 2015.1** | 1.908971 | 1.384527 | 2.632067 | 7.97E-05 | 0.350654 | 98.81734 |
| **Ding et al. 2015.2** | 1.853836 | 1.347382 | 2.550658 | 0.00015 | 0.353824 | 98.82857 |
| **Gokmen et al. 2016** | 1.865358 | 1.337785 | 2.600984 | 0.000237 | 0.373289 | 98.88649 |
| **Kassem et al. 2016** | 1.78968 | 1.311891 | 2.441481 | 0.00024 | 0.332005 | 98.75263 |
| **Assem et al. 2018** | 1.782853 | 1.300125 | 2.444814 | 0.000332 | 0.336488 | 98.76823 |
| **Labenz et al. 2021** | 1.924121 | 1.379763 | 2.683242 | 0.000115 | 0.363199 | 98.21001 |
| **Loosen et al. 2021** | 1.902714 | 1.358104 | 2.665718 | 0.000185 | 0.376314 | 97.94781 |
| **Sheikhi et al. 2022.1** | 1.846282 | 1.322927 | 2.576679 | 0.000312 | 0.373685 | 98.88667 |
| **Sheikhi et al. 2022.2** | 1.834345 | 1.324811 | 2.539849 | 0.000258 | 0.361332 | 98.85178 |
| **Boustany et al. 2023** | 1.581707 | 1.239446 | 2.018479 | 0.000228 | 0.157008 | 93.01054 |
| **Disessa et al. 2023** | 1.824051 | 1.304244 | 2.551028 | 0.000445 | 0.373096 | 98.86722 |

| **Study** | **Estimate** | **CI_lb** | **CI_ub** | **p_value** | **Tau2** | **I2** |
| --- | --- | --- | --- | --- | --- | --- |
| **Pagadala et al. 2011** | 1.973732 | 1.319699 | 2.9519 | 0.000931 | 0.823018 | 96.83991 |
| **Chung et al. 2013.1** | 1.970775 | 1.314334 | 2.955073 | 0.001029 | 0.829709 | 96.77784 |
| **Chung et al. 2013.2** | 2.004306 | 1.332365 | 3.015121 | 0.000846 | 0.843324 | 96.16295 |
| **Posadas-Romero et al. 2014** | 2.056891 | 1.386621 | 3.051159 | 0.000338 | 0.780492 | 96.60887 |
| **Lee et al. 2015.1** | 2.047415 | 1.372634 | 3.053917 | 0.000444 | 0.805986 | 96.71895 |
| **Lee et al. 2015.2** | 2.059343 | 1.387793 | 3.055855 | 0.000334 | 0.78003 | 96.19318 |
| **Bano et al. 2016** | 2.023998 | 1.347015 | 3.041219 | 0.000689 | 0.83816 | 96.53686 |
| **Gokmen et al. 2016** | 2.034276 | 1.362925 | 3.036321 | 0.00051 | 0.813625 | 96.80445 |
| **Kim et al. 2018.1** | 2.015569 | 1.48498 | 2.735738 | 6.9E-06 | 0.429889 | 94.06953 |
| **Kim et al. 2018.2** | 1.97327 | 1.317427 | 2.955605 | 0.000976 | 0.827176 | 96.83561 |
| **Tahara et al. 2019** | 1.938717 | 1.30419 | 2.881961 | 0.001064 | 0.796312 | 96.74259 |
| **Grewal et al. 2020** | 1.887394 | 1.283247 | 2.775971 | 0.001251 | 0.744602 | 96.50139 |
| **Popescu et al. 2020.1** | 1.992881 | 1.328804 | 2.988833 | 0.000854 | 0.833686 | 96.86431 |
| **Popescu et al. 2020.2** | 1.959555 | 1.321706 | 2.905227 | 0.000813 | 0.795259 | 96.75408 |
| **Kim et al. 2020** | 2.006312 | 1.333913 | 3.017654 | 0.000828 | 0.842833 | 96.52563 |
| **Chen et al. 2022** | 1.980829 | 1.318627 | 2.975585 | 0.000994 | 0.836949 | 96.53812 |
| **Patel et al. 2023.1** | 1.832591 | 1.283859 | 2.615855 | 0.000849 | 0.642205 | 96.01746 |
| **Patel et al. 2023.2** | 1.670845 | 1.24569 | 2.241106 | 0.000612 | 0.400694 | 93.75275 |
| **Elshinshawy et al. 2023.1** | 1.844297 | 1.287205 | 2.642493 | 0.00085 | 0.658067 | 96.10977 |
| **Elshinshawy et al. 2023.2** | 1.820826 | 1.280899 | 2.588344 | 0.000839 | 0.625387 | 95.91481 |
| **Fan et al. 2023** | 2.048879 | 1.37164 | 3.0605 | 0.000459 | 0.81032 | 96.17616 |
| **Kouvari et al. 2024** | 1.980987 | 1.321426 | 2.969753 | 0.000936 | 0.831078 | 96.84784 |
| **Bayyigit et al. 2024.1** | 2.04977 | 1.3895 | 3.02379 | 0.000297 | 0.764852 | 96.62108 |
| **Bayyigit et al. 2024.2** | 2.010387 | 1.347553 | 2.999255 | 0.000623 | 0.817223 | 96.8324 |
| **Wang et al. 2024** | 1.988326 | 1.323402 | 2.987332 | 0.000936 | 0.838526 | 96.76485 |

| Study | Estimate | CI_lb | CI_ub | p_value | Tau2 | I2 |
| --- | --- | --- | --- | --- | --- | --- |
| Ding et al. 2015 | 0.05329 | -0.12418 | 0.23076 | 0.556175 | 0.05426 | 87.84578 |
| Eshraghiyan et al. 2013 | 0.125236 | -0.01042 | 0.260893 | 0.070389 | 0.027921 | 79.27386 |
| Gokmen et al. 2016 | 0.086234 | -0.08431 | 0.256782 | 0.321676 | 0.050415 | 88.03713 |
| Assem et al. 2018 | 0.057507 | -0.11653 | 0.231546 | 0.517234 | 0.0534 | 88.8109 |
| Disessa et al. 2023 | 0.032284 | -0.13004 | 0.194611 | 0.696684 | 0.043061 | 82.26629 |
| Kassem et al. 2016 | 0.104215 | -0.0483 | 0.256733 | 0.180494 | 0.039953 | 85.84548 |
| Sheikhi et al. 2022 | 0.080754 | -0.09593 | 0.257436 | 0.370349 | 0.05351 | 87.31367 |
| Bi 2024 | 0.038465 | -0.1285 | 0.205432 | 0.651609 | 0.047064 | 86.5646 |
| Lu et al. 2024 | 0.043273 | -0.12878 | 0.215327 | 0.622048 | 0.050332 | 86.90739 |

| Study | Estimate | CI_lb | CI_ub | p_value | Tau2 | I2 |
| --- | --- | --- | --- | --- | --- | --- |
| Ding et al. 2015 | -1.00703 | -2.5174 | 0.503348 | 0.191285 | 5.176604 | 98.44808 |
| Eshraghiyan et al. 2013 | -0.96229 | -2.47787 | 0.553293 | 0.213338 | 5.221037 | 98.66794 |
| Gokmen et al. 2016 | -0.92205 | -2.4361 | 0.59199 | 0.232627 | 5.21939 | 98.71935 |
| Assem et al. 2018 | -0.96322 | -2.46757 | 0.541141 | 0.209503 | 5.180525 | 98.74846 |
| Disessa et al. 2023 | -1.05358 | -2.53859 | 0.431422 | 0.16436 | 5.035685 | 98.70653 |
| Kassem et al. 2016 | -0.17956 | -0.43681 | 0.077681 | 0.171277 | 0.067859 | 50.13036 |
| Sheikhi et al. 2022 | -0.99524 | -2.50713 | 0.516654 | 0.196984 | 5.19104 | 98.60265 |
| Bi 2024 | -1.06125 | -2.55355 | 0.431056 | 0.163371 | 5.050683 | 98.47814 |
| Kim et al. 2024 | -1.03473 | -2.53794 | 0.468489 | 0.177297 | 5.12372 | 98.17175 |
| Lu et al. 2024 | -1.07213 | -2.55918 | 0.414931 | 0.157633 | 5.015765 | 98.53079 |

| **Study** | **Estimate** | **CI_lb** | **CI_ub** | **p_value** | **Tau2** | **I2** |
| --- | --- | --- | --- | --- | --- | --- |
| **Ding et al. 2015** | 0.544104 | 0.056302 | 1.031906 | 0.028802 | 0.692073 | 98.05536 |
| **Eshraghiyan et al. 2013** | 0.61093 | 0.147953 | 1.073906 | 0.009701 | 0.618573 | 97.82093 |
| **Lee et al. 2015** | 0.594459 | 0.115399 | 1.073519 | 0.015012 | 0.664815 | 97.80972 |
| **Ludwig et al. 2015** | 0.593641 | 0.114922 | 1.072361 | 0.015079 | 0.664777 | 97.97996 |
| **Sheikhi et al. 2022** | 0.396757 | 0.025939 | 0.767574 | 0.035988 | 0.400617 | 96.88936 |
| **Gokmen et al. 2016** | 0.516259 | 0.076754 | 0.955764 | 0.021321 | 0.60183 | 97.91328 |
| **Assem et al. 2018** | 0.483481 | 0.032662 | 0.9343 | 0.035556 | 0.600093 | 97.89806 |
| **Disessa et al. 2023** | 0.57663 | 0.089266 | 1.063994 | 0.020398 | 0.689374 | 97.54069 |
| **Kassem et al. 2016** | 0.285223 | 0.031631 | 0.538815 | 0.027494 | 0.165151 | 92.59758 |
| **Kaltenbach et al. 2016** | 0.570933 | 0.083509 | 1.058357 | 0.02169 | 0.691125 | 98.06647 |
| **Wang et al. 2024** | 0.545883 | 0.058288 | 1.033478 | 0.028217 | 0.69206 | 98.08999 |
| **Bi 2024** | 0.593576 | 0.114884 | 1.072269 | 0.015084 | 0.664773 | 97.9856 |
| **Kim et al. 2024** | 0.597688 | 0.120401 | 1.074975 | 0.014113 | 0.65903 | 97.30345 |
| **Lu et al. 2024** | 0.586815 | 0.103972 | 1.069658 | 0.017218 | 0.67667 | 97.96944 |

1. Bayyigit, A., et al., *Hypothyroidism and subclinical hypothyroidism are associated with fatty pancreas (Non-Alcoholic Fatty Pancreas Disease).* DIABETES-METABOLISM RESEARCH AND REVIEWS, 2024. **40**(2).

2. Bi, T., *Relationship between thyroid hormone levels and metabolic dysfunction associated steatotic liver disease in patients with type 2 diabetes: A clinical study.* Medicine (Baltimore), 2024. **103**(26): p. e38643.

3. Kouvari, M., et al., *Thyroid function, adipokines and mitokines in metabolic dysfunction-associated steatohepatitis: A multi-centre biopsy-based observational study.* Liver Int, 2024. **44**(3): p. 848-864.

4. Mahashabde, M.L., et al., *A Study of Non-alcoholic Fatty Liver Disease in Patients With Hypothyroidism: A Cross-Sectional Study in a Tertiary Care Hospital.* Cureus, 2024. **16**(9): p. e68956.

5. Elshinshawy, S., et al., *The Interrelation Between Hypothyroidism and Non-alcoholic Fatty Liver Disease, a Cross-sectional Study.* J Clin Exp Hepatol, 2023. **13**(4): p. 638-648.

6. Patel, M., S. Acharya, and S. Kumar, *Prevalence of Nonalcoholic Fatty Liver Disease in Hypothyroid Subjects: A Cross-sectional Comparative Study.* INDIAN JOURNAL OF MEDICAL SPECIALITIES, 2023. **14**(3): p. 145-151.

7. Chen, S., et al., *Relationship Between Thyroid Hormone and Liver Steatosis Analysis Parameter in Obese Participants: A Case-Control Study.* Diabetes, Metabolic Syndrome and Obesity, 2022. **15**: p. 887-896.

8. Sheikhi, V. and Z. Heidari, *Association of Subclinical Hypothyroidism with Nonalcoholic Fatty Liver Disease in Patients with Type 2 Diabetes Mellitus: A Cross-Sectional Study.* Adv Biomed Res, 2022. **11**: p. 124.

9. Grewal, H., et al., *Non-alcoholic fatty liver disease in patients with hypothyroidism presenting at a rural tertiary care centre in north India.* Tropical Doctor, 2021. **51**(2): p. 181-184.

10. Tahara, K., et al., *Thyroid-stimulating hormone is an independent risk factor of non-alcoholic fatty liver disease.* JGH Open, 2020. **4**(3): p. 400-404.

11. Hussein, M.A., et al., *Thyroid dysfunction and insulin resistance in patients with nonalcoholic fatty liver disease.* The Egyptian Journal of Internal Medicine, 2018. **30**(3): p. 97-102.

12. Kaltenbach, T.E., et al., *Thyroid dysfunction and hepatic steatosis in overweight children and adolescents.* Pediatr Obes, 2017. **12**(1): p. 67-74.

13. Ding, W.J., et al., *Thyroid function is associated with non-alcoholic fatty liver disease in chronic hepatitis B-infected subjects.* J Gastroenterol Hepatol, 2015. **30**(12): p. 1753-8.

14. Ludwig, U., et al., *Subclinical and clinical hypothyroidism and non-alcoholic fatty liver disease: a cross-sectional study of a random population sample aged 18 to 65 years.* BMC Endocr Disord, 2015. **15**: p. 41.

15. Posadas-Romero, C., et al., *Fatty liver largely explains associations of subclinical hypothyroidism with insulin resistance, metabolic syndrome, and subclinical coronary atherosclerosis.* Eur J Endocrinol, 2014. **171**(3): p. 319-25.

16. Chung, G.E., et al., *Non-alcoholic fatty liver disease across the spectrum of hypothyroidism.* J Hepatol, 2012. **57**(1): p. 150-6.

17. Eshraghian, A., et al., *Nonalcoholic fatty liver disease in a cluster of Iranian population: thyroid status and metabolic risk factors.* Arch Iran Med, 2013. **16**(10): p. 584-9.

18. Kim, H.I., et al., *Triiodothyronine Is Associated with Incidence/Resolution of Steatotic Liver Disease: Longitudinal Study in Euthyroid Korean.* Endocrinol Metab (Seoul), 2024.

19. Lu, W., et al., *Associations of sex-related and thyroid-related hormones with risk of metabolic dysfunction-associated fatty liver disease in T2DM patients.* BMC Endocr Disord, 2024. **24**(1): p. 84.

20. Wang, S., et al., *Low thyroid function is associated with metabolic dysfunction-associated steatotic liver disease.* JGH OPEN, 2024. **8**(2).

21. Boustany, A., et al., *Non-alcoholic steatohepatitis is independently associated with a history of gestational diabetes mellitus.* J Gastroenterol Hepatol, 2023. **38**(6): p. 984-988.

22. Di Sessa, A., et al., *Association between non-alcoholic fatty liver disease and subclinical hypothyroidism in children with obesity.* JOURNAL OF ENDOCRINOLOGICAL INVESTIGATION, 2023. **46**(9): p. 1835-1842.

23. Fan, H., et al., *Low thyroid function is associated with an increased risk of advanced fibrosis in patients with metabolic dysfunction-associated fatty liver disease.* BMC GASTROENTEROLOGY, 2023. **23**(1).

24. Loosen, S.H., et al., *Incidences of hypothyroidism and autoimmune thyroiditis are increased in patients with nonalcoholic fatty liver disease.* Eur J Gastroenterol Hepatol, 2021. **33**(1S Suppl 1): p. e1008-e1012.

25. Kim, D., et al., *Low Thyroid Function in Nonalcoholic Fatty Liver Disease Is an Independent Predictor of All-Cause and Cardiovascular Mortality.* Am J Gastroenterol, 2020. **115**(9): p. 1496-1504.

26. Kim, D., et al., *Subclinical Hypothyroidism and Low-Normal Thyroid Function Are Associated With Nonalcoholic Steatohepatitis and Fibrosis.* Clin Gastroenterol Hepatol, 2018. **16**(1): p. 123-131.e1.

27. Bano, A., et al., *Thyroid Function and the Risk of Nonalcoholic Fatty Liver Disease: The Rotterdam Study.* J Clin Endocrinol Metab, 2016. **101**(8): p. 3204-11.

28. Lee, K.W., et al., *Impact of hypothyroidism on the development of non-alcoholic fatty liver disease: A 4-year retrospective cohort study.* Clin Mol Hepatol, 2015. **21**(4): p. 372-8.

29. Labenz, C., et al., *Impact of thyroid disorders on the incidence of non-alcoholic fatty liver disease in Germany.* United European Gastroenterol J, 2021. **9**(7): p. 829-836.

30. Popescu, M., et al., *Hypothyroidism-A Risk Factor for the Non-Alcoholic Fatty Liver Disease.* Res. & Sci. Today, 2020. **20**: p. 139.

31. Gökmen, F.Y., et al., *FT3/FT4 ratio predicts non-alcoholic fatty liver disease independent of metabolic parameters in patients with euthyroidism and hypothyroidism.* Clinics (Sao Paulo), 2016. **71**(4): p. 221-5.

32. Kassem¹, A., et al., *Association and impact of non-alcoholic fatty liver disease on thyroid function.* Int. J. Curr. Res. Med. Sci, 2017. **3**(7): p. 94-107.

33. Parikh, P., A. Phadke, and P. Sawant, *Prevalence of hypothyroidism in nonalcoholic fatty liver disease in patients attending a tertiary hospital in western India.* Indian J Gastroenterol, 2015. **34**(2): p. 169-73.

34. Pagadala, M.R., et al., *Prevalence of hypothyroidism in nonalcoholic fatty liver disease.* Dig Dis Sci, 2012. **57**(2): p. 528-34.

35. Liangpunsakul, S. and N. Chalasani, *Is hypothyroidism a risk factor for non-alcoholic steatohepatitis?* J Clin Gastroenterol, 2003. **37**(4): p. 340-3.
