# Supplementary material for: The reciprocal relationship between non-alcoholic fatty liver disease and hypothyroidism: A systematic review and meta-analysis of about 39 million individuals
Source: PLoS One. 2025 Dec 18;20(12):e0338413. doi: 10.1371/journal.pone.0338413 (PMC12714247; doi:10.1371/journal.pone.0338413)
Supplement: S2 Table — (DOCX) [file pone.0338413.s018.docx]

| Author | 1 | 2 | 3 | 4 | 5 | 6 | 7 | 8 | 9 | 10 | 11 |
| --- | --- | --- | --- | --- | --- | --- | --- | --- | --- | --- | --- |
| **Kim et al. 2024 [1]** | Yes | Yes | Yes | Yes | Yes | Yes | No | Yes | Yes | No | Yes |
| **Lu et al. 2024 [2]** | Unclear | Yes | Yes | Yes | Yes | Yes | Yes | No | No | No | Yes |
| **Wang et al. 2024 [3]** | Yes | Yes | Yes | Yes | Yes | Yes | Yes | Yes | No | NA | Yes |
| **Boustany et al. 2023 [4]** | Yes | Yes | Yes | Yes | Yes | Yes | Yes | NA | NA | NA | Yes |
| **Di Sessa et al. 2023 [5]** | Yes | Yes | Yes | Yes | Yes | Unclear | Yes | NA | NA | NA | Yes |
| **Fan et al. 2023 [6]** | Yes | Yes | Yes | Yes | Yes | Unclear | Yes | No | NA | NA | Yes |
| **Loosen et al. 2021 [7]** | Yes | Yes | Unclear | Yes | Yes | Yes | Unclear | Yes | Unclear | Unclear | Yes |
| **Kim et al. 2020 [8]** | Yes | Yes | Yes | Yes | Yes | No | Yes | Yes | No | Yes | Yes |
| **Kim et al. 2018 [9]** | Yes | Yes | Yes | Yes | Yes | No | Yes | No | Unclear | Unclear | Yes |
| **Bano et al. 2016 [10]** | Yes | Yes | Yes | Yes | Yes | Unclear | Unclear | Yes | Unclear | NA | Yes |
| **Lee et al. 2015 [11]** | Yes | Yes | Yes | Yes | Yes | Yes | Unclear | Yes | No | NA | Yes |

1. Kim, H.I., et al., *Triiodothyronine Is Associated with Incidence/Resolution of Steatotic Liver Disease: Longitudinal Study in Euthyroid Korean.* Endocrinol Metab (Seoul), 2024.

2. Lu, W., et al., *Associations of sex-related and thyroid-related hormones with risk of metabolic dysfunction-associated fatty liver disease in T2DM patients.* BMC Endocr Disord, 2024. **24**(1): p. 84.

3. Wang, S., et al., *Low thyroid function is associated with metabolic dysfunction-associated steatotic liver disease.* JGH OPEN, 2024. **8**(2).

4. Boustany, A., et al., *Non-alcoholic steatohepatitis is independently associated with a history of gestational diabetes mellitus.* J Gastroenterol Hepatol, 2023. **38**(6): p. 984-988.

5. Di Sessa, A., et al., *Association between non-alcoholic fatty liver disease and subclinical hypothyroidism in children with obesity.* JOURNAL OF ENDOCRINOLOGICAL INVESTIGATION, 2023. **46**(9): p. 1835-1842.

6. Fan, H., et al., *Low thyroid function is associated with an increased risk of advanced fibrosis in patients with metabolic dysfunction-associated fatty liver disease.* BMC GASTROENTEROLOGY, 2023. **23**(1).

7. Loosen, S.H., et al., *Incidences of hypothyroidism and autoimmune thyroiditis are increased in patients with nonalcoholic fatty liver disease.* Eur J Gastroenterol Hepatol, 2021. **33**(1S Suppl 1): p. e1008-e1012.

8. Kim, D., et al., *Low Thyroid Function in Nonalcoholic Fatty Liver Disease Is an Independent Predictor of All-Cause and Cardiovascular Mortality.* Am J Gastroenterol, 2020. **115**(9): p. 1496-1504.

9. Kim, D., et al., *Subclinical Hypothyroidism and Low-Normal Thyroid Function Are Associated With Nonalcoholic Steatohepatitis and Fibrosis.* Clin Gastroenterol Hepatol, 2018. **16**(1): p. 123-131.e1.

10. Bano, A., et al., *Thyroid Function and the Risk of Nonalcoholic Fatty Liver Disease: The Rotterdam Study.* J Clin Endocrinol Metab, 2016. **101**(8): p. 3204-11.

11. Lee, K.W., et al., *Impact of hypothyroidism on the development of non-alcoholic fatty liver disease: A 4-year retrospective cohort study.* Clin Mol Hepatol, 2015. **21**(4): p. 372-8.
