# Supplementary material for: The reciprocal relationship between non-alcoholic fatty liver disease and hypothyroidism: A systematic review and meta-analysis of about 39 million individuals
Source: PLoS One. 2025 Dec 18;20(12):e0338413. doi: 10.1371/journal.pone.0338413 (PMC12714247; doi:10.1371/journal.pone.0338413)
Supplement: S3 Table — (DOCX) [file pone.0338413.s019.docx]

| Author | 1 | 2 | 3 | 4 | 5 | 6 | 7 | 8 | 9 | 10 |
| --- | --- | --- | --- | --- | --- | --- | --- | --- | --- | --- |
| **Labenz et al 2021 [1]** | Yes | Yes | Yes | Yes | Yes | Yes | Yes | Yes | Yes | Yes |
| **Popescu et al. 2020 [2]** | Yes | No | Yes | Yes | Yes | Yes | No | Yes | No | No |
| **Gokmen et al. 2016 [3]** | Yes | Unclear | Unclear | Yes | Yes | Yes | Yes | Yes | Unclear | Yes |
| **Kassem et al. 2016 [4]** | Yes | No | No | Yes | Yes | Yes | Yes | Yes | Unclear | Unclear |
| **Parikh et al. 2015 [5]** | No | Unclear | Yes | Unclear | Yes | Yes | Yes | Yes | Unclear | Yes |
| **Pagadala et al. 2011 [6]** | Yes | Yes | Yes | Unclear | Yes | Yes | Yes | Yes | Unclear | Yes |
| **Liangpunsakul et al. 2003 [7]** | Yes | Yes | Yes | Unclear | Unclear | Yes | Yes | Yes | Unclear | Yes |

1. Labenz, C., et al., *Impact of thyroid disorders on the incidence of non-alcoholic fatty liver disease in Germany.* United European Gastroenterol J, 2021. **9**(7): p. 829-836.

2. Popescu, M., et al., *Hypothyroidism-A Risk Factor for the Non-Alcoholic Fatty Liver Disease.* Res. & Sci. Today, 2020. **20**: p. 139.

3. Gökmen, F.Y., et al., *FT3/FT4 ratio predicts non-alcoholic fatty liver disease independent of metabolic parameters in patients with euthyroidism and hypothyroidism.* Clinics (Sao Paulo), 2016. **71**(4): p. 221-5.

4. Kassem¹, A., et al., *Association and impact of non-alcoholic fatty liver disease on thyroid function.* Int. J. Curr. Res. Med. Sci, 2017. **3**(7): p. 94-107.

5. Parikh, P., A. Phadke, and P. Sawant, *Prevalence of hypothyroidism in nonalcoholic fatty liver disease in patients attending a tertiary hospital in western India.* Indian J Gastroenterol, 2015. **34**(2): p. 169-73.

6. Pagadala, M.R., et al., *Prevalence of hypothyroidism in nonalcoholic fatty liver disease.* Dig Dis Sci, 2012. **57**(2): p. 528-34.

7. Liangpunsakul, S. and N. Chalasani, *Is hypothyroidism a risk factor for non-alcoholic steatohepatitis?* J Clin Gastroenterol, 2003. **37**(4): p. 340-3.
