# Supplementary material for: The reciprocal relationship between non-alcoholic fatty liver disease and hypothyroidism: A systematic review and meta-analysis of about 39 million individuals
Source: PLoS One. 2025 Dec 18;20(12):e0338413. doi: 10.1371/journal.pone.0338413 (PMC12714247; doi:10.1371/journal.pone.0338413)
Supplement: S4 Table — (DOCX) [file pone.0338413.s020.docx]

| **Study** | **Estimate** | **CI_lb** | **CI_ub** | **p_value** | **Tau2** | **I2** |
| --- | --- | --- | --- | --- | --- | --- |
| **Liangpunsakul et al. 2003** | 1.833019 | 1.313228 | 2.558549 | 0.000369 | 0.372176 | 98.88072 |
| **Padagala et al. 2011** | 1.81556 | 1.302217 | 2.531267 | 0.000436 | 0.367091 | 98.86275 |
| **Eshraghiyan et al. 2013.1** | 1.901517 | 1.367499 | 2.644074 | 0.000133 | 0.365315 | 98.86237 |
| **Eshraghiyan et al. 2013.2** | 1.902246 | 1.370049 | 2.641176 | 0.000123 | 0.362923 | 98.85555 |
| **Ludwig et al. 2015** | 1.931768 | 1.396501 | 2.672199 | 6.97E-05 | 0.347892 | 98.80413 |
| **Parikh et al. 2015.1** | 1.768455 | 1.302439 | 2.401214 | 0.000259 | 0.319306 | 98.70366 |
| **Parikh et al. 2015.2** | 1.803598 | 1.306584 | 2.489671 | 0.000336 | 0.351115 | 98.81848 |
| **Lee et al. 2015.1** | 1.959424 | 1.42685 | 2.690781 | 3.23E-05 | 0.322052 | 98.65574 |
| **Lee et al. 2015.2** | 1.936944 | 1.399573 | 2.680639 | 6.67E-05 | 0.346339 | 98.79455 |
| **Ding et al. 2015.1** | 1.908971 | 1.384527 | 2.632067 | 7.97E-05 | 0.350654 | 98.81734 |
| **Ding et al. 2015.2** | 1.853836 | 1.347382 | 2.550658 | 0.00015 | 0.353824 | 98.82857 |
| **Gokmen et al. 2016** | 1.865358 | 1.337785 | 2.600984 | 0.000237 | 0.373289 | 98.88649 |
| **Kassem et al. 2016** | 1.78968 | 1.311891 | 2.441481 | 0.00024 | 0.332005 | 98.75263 |
| **Assem et al. 2018** | 1.782853 | 1.300125 | 2.444814 | 0.000332 | 0.336488 | 98.76823 |
| **Labenz et al. 2021** | 1.924121 | 1.379763 | 2.683242 | 0.000115 | 0.363199 | 98.21001 |
| **Loosen et al. 2021** | 1.902714 | 1.358104 | 2.665718 | 0.000185 | 0.376314 | 97.94781 |
| **Sheikhi et al. 2022.1** | 1.846282 | 1.322927 | 2.576679 | 0.000312 | 0.373685 | 98.88667 |
| **Sheikhi et al. 2022.2** | 1.834345 | 1.324811 | 2.539849 | 0.000258 | 0.361332 | 98.85178 |
| **Boustany et al. 2023** | 1.581707 | 1.239446 | 2.018479 | 0.000228 | 0.157008 | 93.01054 |
| **Disessa et al. 2023** | 1.824051 | 1.304244 | 2.551028 | 0.000445 | 0.373096 | 98.86722 |

1. Bayyigit, A., et al., *Hypothyroidism and subclinical hypothyroidism are associated with fatty pancreas (Non-Alcoholic Fatty Pancreas Disease).* DIABETES-METABOLISM RESEARCH AND REVIEWS, 2024. **40**(2).

2. Bi, T., *Relationship between thyroid hormone levels and metabolic dysfunction associated steatotic liver disease in patients with type 2 diabetes: A clinical study.* Medicine (Baltimore), 2024. **103**(26): p. e38643.

3. Kouvari, M., et al., *Thyroid function, adipokines and mitokines in metabolic dysfunction-associated steatohepatitis: A multi-centre biopsy-based observational study.* Liver Int, 2024. **44**(3): p. 848-864.

4. Mahashabde, M.L., et al., *A Study of Non-alcoholic Fatty Liver Disease in Patients With Hypothyroidism: A Cross-Sectional Study in a Tertiary Care Hospital.* Cureus, 2024. **16**(9): p. e68956.

5. Elshinshawy, S., et al., *The Interrelation Between Hypothyroidism and Non-alcoholic Fatty Liver Disease, a Cross-sectional Study.* J Clin Exp Hepatol, 2023. **13**(4): p. 638-648.

6. Patel, M., S. Acharya, and S. Kumar, *Prevalence of Nonalcoholic Fatty Liver Disease in Hypothyroid Subjects: A Cross-sectional Comparative Study.* INDIAN JOURNAL OF MEDICAL SPECIALITIES, 2023. **14**(3): p. 145-151.

7. Chen, S., et al., *Relationship Between Thyroid Hormone and Liver Steatosis Analysis Parameter in Obese Participants: A Case-Control Study.* Diabetes, Metabolic Syndrome and Obesity, 2022. **15**: p. 887-896.

8. Sheikhi, V. and Z. Heidari, *Association of Subclinical Hypothyroidism with Nonalcoholic Fatty Liver Disease in Patients with Type 2 Diabetes Mellitus: A Cross-Sectional Study.* Adv Biomed Res, 2022. **11**: p. 124.

9. Grewal, H., et al., *Non-alcoholic fatty liver disease in patients with hypothyroidism presenting at a rural tertiary care centre in north India.* Tropical Doctor, 2021. **51**(2): p. 181-184.

10. Tahara, K., et al., *Thyroid-stimulating hormone is an independent risk factor of non-alcoholic fatty liver disease.* JGH Open, 2020. **4**(3): p. 400-404.

11. Hussein, M.A., et al., *Thyroid dysfunction and insulin resistance in patients with nonalcoholic fatty liver disease.* The Egyptian Journal of Internal Medicine, 2018. **30**(3): p. 97-102.

12. Kaltenbach, T.E., et al., *Thyroid dysfunction and hepatic steatosis in overweight children and adolescents.* Pediatr Obes, 2017. **12**(1): p. 67-74.

13. Ding, W.J., et al., *Thyroid function is associated with non-alcoholic fatty liver disease in chronic hepatitis B-infected subjects.* J Gastroenterol Hepatol, 2015. **30**(12): p. 1753-8.

14. Ludwig, U., et al., *Subclinical and clinical hypothyroidism and non-alcoholic fatty liver disease: a cross-sectional study of a random population sample aged 18 to 65 years.* BMC Endocr Disord, 2015. **15**: p. 41.

15. Posadas-Romero, C., et al., *Fatty liver largely explains associations of subclinical hypothyroidism with insulin resistance, metabolic syndrome, and subclinical coronary atherosclerosis.* Eur J Endocrinol, 2014. **171**(3): p. 319-25.

16. Chung, G.E., et al., *Non-alcoholic fatty liver disease across the spectrum of hypothyroidism.* J Hepatol, 2012. **57**(1): p. 150-6.

17. Eshraghian, A., et al., *Nonalcoholic fatty liver disease in a cluster of Iranian population: thyroid status and metabolic risk factors.* Arch Iran Med, 2013. **16**(10): p. 584-9.

18. Kim, H.I., et al., *Triiodothyronine Is Associated with Incidence/Resolution of Steatotic Liver Disease: Longitudinal Study in Euthyroid Korean.* Endocrinol Metab (Seoul), 2024.

19. Lu, W., et al., *Associations of sex-related and thyroid-related hormones with risk of metabolic dysfunction-associated fatty liver disease in T2DM patients.* BMC Endocr Disord, 2024. **24**(1): p. 84.

20. Wang, S., et al., *Low thyroid function is associated with metabolic dysfunction-associated steatotic liver disease.* JGH OPEN, 2024. **8**(2).

21. Boustany, A., et al., *Non-alcoholic steatohepatitis is independently associated with a history of gestational diabetes mellitus.* J Gastroenterol Hepatol, 2023. **38**(6): p. 984-988.

22. Di Sessa, A., et al., *Association between non-alcoholic fatty liver disease and subclinical hypothyroidism in children with obesity.* JOURNAL OF ENDOCRINOLOGICAL INVESTIGATION, 2023. **46**(9): p. 1835-1842.

23. Fan, H., et al., *Low thyroid function is associated with an increased risk of advanced fibrosis in patients with metabolic dysfunction-associated fatty liver disease.* BMC GASTROENTEROLOGY, 2023. **23**(1).

24. Loosen, S.H., et al., *Incidences of hypothyroidism and autoimmune thyroiditis are increased in patients with nonalcoholic fatty liver disease.* Eur J Gastroenterol Hepatol, 2021. **33**(1S Suppl 1): p. e1008-e1012.

25. Kim, D., et al., *Low Thyroid Function in Nonalcoholic Fatty Liver Disease Is an Independent Predictor of All-Cause and Cardiovascular Mortality.* Am J Gastroenterol, 2020. **115**(9): p. 1496-1504.

26. Kim, D., et al., *Subclinical Hypothyroidism and Low-Normal Thyroid Function Are Associated With Nonalcoholic Steatohepatitis and Fibrosis.* Clin Gastroenterol Hepatol, 2018. **16**(1): p. 123-131.e1.

27. Bano, A., et al., *Thyroid Function and the Risk of Nonalcoholic Fatty Liver Disease: The Rotterdam Study.* J Clin Endocrinol Metab, 2016. **101**(8): p. 3204-11.

28. Lee, K.W., et al., *Impact of hypothyroidism on the development of non-alcoholic fatty liver disease: A 4-year retrospective cohort study.* Clin Mol Hepatol, 2015. **21**(4): p. 372-8.

29. Labenz, C., et al., *Impact of thyroid disorders on the incidence of non-alcoholic fatty liver disease in Germany.* United European Gastroenterol J, 2021. **9**(7): p. 829-836.

30. Popescu, M., et al., *Hypothyroidism-A Risk Factor for the Non-Alcoholic Fatty Liver Disease.* Res. & Sci. Today, 2020. **20**: p. 139.

31. Gökmen, F.Y., et al., *FT3/FT4 ratio predicts non-alcoholic fatty liver disease independent of metabolic parameters in patients with euthyroidism and hypothyroidism.* Clinics (Sao Paulo), 2016. **71**(4): p. 221-5.

32. Kassem¹, A., et al., *Association and impact of non-alcoholic fatty liver disease on thyroid function.* Int. J. Curr. Res. Med. Sci, 2017. **3**(7): p. 94-107.

33. Parikh, P., A. Phadke, and P. Sawant, *Prevalence of hypothyroidism in nonalcoholic fatty liver disease in patients attending a tertiary hospital in western India.* Indian J Gastroenterol, 2015. **34**(2): p. 169-73.

34. Pagadala, M.R., et al., *Prevalence of hypothyroidism in nonalcoholic fatty liver disease.* Dig Dis Sci, 2012. **57**(2): p. 528-34.

35. Liangpunsakul, S. and N. Chalasani, *Is hypothyroidism a risk factor for non-alcoholic steatohepatitis?* J Clin Gastroenterol, 2003. **37**(4): p. 340-3.
