# Supplementary material for: The reciprocal relationship between non-alcoholic fatty liver disease and hypothyroidism: A systematic review and meta-analysis of about 39 million individuals
Source: PLoS One. 2025 Dec 18;20(12):e0338413. doi: 10.1371/journal.pone.0338413 (PMC12714247; doi:10.1371/journal.pone.0338413)
Supplement: S5 Table — (DOCX) [file pone.0338413.s021.docx]

| **Study** | **Estimate** | **CI_lb** | **CI_ub** | **p_value** | **Tau2** | **I2** |
| --- | --- | --- | --- | --- | --- | --- |
| **Pagadala et al. 2011** | 1.973732 | 1.319699 | 2.9519 | 0.000931 | 0.823018 | 96.83991 |
| **Chung et al. 2013.1** | 1.970775 | 1.314334 | 2.955073 | 0.001029 | 0.829709 | 96.77784 |
| **Chung et al. 2013.2** | 2.004306 | 1.332365 | 3.015121 | 0.000846 | 0.843324 | 96.16295 |
| **Posadas-Romero et al. 2014** | 2.056891 | 1.386621 | 3.051159 | 0.000338 | 0.780492 | 96.60887 |
| **Lee et al. 2015.1** | 2.047415 | 1.372634 | 3.053917 | 0.000444 | 0.805986 | 96.71895 |
| **Lee et al. 2015.2** | 2.059343 | 1.387793 | 3.055855 | 0.000334 | 0.78003 | 96.19318 |
| **Bano et al. 2016** | 2.023998 | 1.347015 | 3.041219 | 0.000689 | 0.83816 | 96.53686 |
| **Gokmen et al. 2016** | 2.034276 | 1.362925 | 3.036321 | 0.00051 | 0.813625 | 96.80445 |
| **Kim et al. 2018.1** | 2.015569 | 1.48498 | 2.735738 | 6.9E-06 | 0.429889 | 94.06953 |
| **Kim et al. 2018.2** | 1.97327 | 1.317427 | 2.955605 | 0.000976 | 0.827176 | 96.83561 |
| **Tahara et al. 2019** | 1.938717 | 1.30419 | 2.881961 | 0.001064 | 0.796312 | 96.74259 |
| **Grewal et al. 2020** | 1.887394 | 1.283247 | 2.775971 | 0.001251 | 0.744602 | 96.50139 |
| **Popescu et al. 2020.1** | 1.992881 | 1.328804 | 2.988833 | 0.000854 | 0.833686 | 96.86431 |
| **Popescu et al. 2020.2** | 1.959555 | 1.321706 | 2.905227 | 0.000813 | 0.795259 | 96.75408 |
| **Kim et al. 2020** | 2.006312 | 1.333913 | 3.017654 | 0.000828 | 0.842833 | 96.52563 |
| **Chen et al. 2022** | 1.980829 | 1.318627 | 2.975585 | 0.000994 | 0.836949 | 96.53812 |
| **Patel et al. 2023.1** | 1.832591 | 1.283859 | 2.615855 | 0.000849 | 0.642205 | 96.01746 |
| **Patel et al. 2023.2** | 1.670845 | 1.24569 | 2.241106 | 0.000612 | 0.400694 | 93.75275 |
| **Elshinshawy et al. 2023.1** | 1.844297 | 1.287205 | 2.642493 | 0.00085 | 0.658067 | 96.10977 |
| **Elshinshawy et al. 2023.2** | 1.820826 | 1.280899 | 2.588344 | 0.000839 | 0.625387 | 95.91481 |
| **Fan et al. 2023** | 2.048879 | 1.37164 | 3.0605 | 0.000459 | 0.81032 | 96.17616 |
| **Kouvari et al. 2024** | 1.980987 | 1.321426 | 2.969753 | 0.000936 | 0.831078 | 96.84784 |
| **Bayyigit et al. 2024.1** | 2.04977 | 1.3895 | 3.02379 | 0.000297 | 0.764852 | 96.62108 |
| **Bayyigit et al. 2024.2** | 2.010387 | 1.347553 | 2.999255 | 0.000623 | 0.817223 | 96.8324 |
| **Wang et al. 2024** | 1.988326 | 1.323402 | 2.987332 | 0.000936 | 0.838526 | 96.76485 |
