# Supplementary material for: The reciprocal relationship between non-alcoholic fatty liver disease and hypothyroidism: A systematic review and meta-analysis of about 39 million individuals
Source: PLoS One. 2025 Dec 18;20(12):e0338413. doi: 10.1371/journal.pone.0338413 (PMC12714247; doi:10.1371/journal.pone.0338413)
Supplement: S6 Table — (DOCX) [file pone.0338413.s022.docx]

| Study | Estimate | CI_lb | CI_ub | p_value | Tau2 | I2 |
| --- | --- | --- | --- | --- | --- | --- |
| Ding et al. 2015 | 0.05329 | -0.12418 | 0.23076 | 0.556175 | 0.05426 | 87.84578 |
| Eshraghiyan et al. 2013 | 0.125236 | -0.01042 | 0.260893 | 0.070389 | 0.027921 | 79.27386 |
| Gokmen et al. 2016 | 0.086234 | -0.08431 | 0.256782 | 0.321676 | 0.050415 | 88.03713 |
| Assem et al. 2018 | 0.057507 | -0.11653 | 0.231546 | 0.517234 | 0.0534 | 88.8109 |
| Disessa et al. 2023 | 0.032284 | -0.13004 | 0.194611 | 0.696684 | 0.043061 | 82.26629 |
| Kassem et al. 2016 | 0.104215 | -0.0483 | 0.256733 | 0.180494 | 0.039953 | 85.84548 |
| Sheikhi et al. 2022 | 0.080754 | -0.09593 | 0.257436 | 0.370349 | 0.05351 | 87.31367 |
| Bi 2024 | 0.038465 | -0.1285 | 0.205432 | 0.651609 | 0.047064 | 86.5646 |
| Lu et al. 2024 | 0.043273 | -0.12878 | 0.215327 | 0.622048 | 0.050332 | 86.90739 |
