# Supplementary material for: The reciprocal relationship between non-alcoholic fatty liver disease and hypothyroidism: A systematic review and meta-analysis of about 39 million individuals
Source: PLoS One. 2025 Dec 18;20(12):e0338413. doi: 10.1371/journal.pone.0338413 (PMC12714247; doi:10.1371/journal.pone.0338413)
Supplement: S7 Table — (DOCX) [file pone.0338413.s023.docx]

| Study | Estimate | CI_lb | CI_ub | p_value | Tau2 | I2 |
| --- | --- | --- | --- | --- | --- | --- |
| Ding et al. 2015 | -1.00703 | -2.5174 | 0.503348 | 0.191285 | 5.176604 | 98.44808 |
| Eshraghiyan et al. 2013 | -0.96229 | -2.47787 | 0.553293 | 0.213338 | 5.221037 | 98.66794 |
| Gokmen et al. 2016 | -0.92205 | -2.4361 | 0.59199 | 0.232627 | 5.21939 | 98.71935 |
| Assem et al. 2018 | -0.96322 | -2.46757 | 0.541141 | 0.209503 | 5.180525 | 98.74846 |
| Disessa et al. 2023 | -1.05358 | -2.53859 | 0.431422 | 0.16436 | 5.035685 | 98.70653 |
| Kassem et al. 2016 | -0.17956 | -0.43681 | 0.077681 | 0.171277 | 0.067859 | 50.13036 |
| Sheikhi et al. 2022 | -0.99524 | -2.50713 | 0.516654 | 0.196984 | 5.19104 | 98.60265 |
| Bi 2024 | -1.06125 | -2.55355 | 0.431056 | 0.163371 | 5.050683 | 98.47814 |
| Kim et al. 2024 | -1.03473 | -2.53794 | 0.468489 | 0.177297 | 5.12372 | 98.17175 |
| Lu et al. 2024 | -1.07213 | -2.55918 | 0.414931 | 0.157633 | 5.015765 | 98.53079 |
